# UVB-Induced necroptosis of the skin cells via RIPK3-MLKL activation independent of RIPK1 kinase activity

**DOI:** 10.1038/s41420-025-02471-3

**Published:** 2025-04-12

**Authors:** Tairan Hu, Xiaodong Lai, Li Li, Yi Li, Meng Wang, Haini Zhang, Yan Yang, Chong Zhang, Yan Yan, Baoxi Wang

**Affiliations:** Department of Dermatology, Plastic Surgery Hospital, Chinese Academy of Medical Sciences and Peking Union Medical College, Beijing, China

**Keywords:** Necroptosis, Apoptosis, Kinases

## Abstract

Ultraviolet B (UVB) is recognized for inducing inflammation and death of keratinocytes through the activation of death receptors and pattern recognition receptors (PRRs). Receptor-interacting protein kinase 1 (RIPK1) and RIPK3 play pivotal roles in mediating necroptosis, which can be triggered by the activation of specific death receptors and PRRs. In this study, we observed a reduction of RIPK1 protein after UVB exposure which led to activation of Nuclear factor-kappa B (NF-κB) in HaCaT cells. This activation, in turn, promoted the production of IL-1β and TNF-α. However, RIPK1 kinase remained inactive and did not participate in cell death. Interestingly, UVB radiation triggered the activation of RIPK3 independently of RIPK1 kinase activity and subsequently induced phosphorylation of mixed-lineage kinase domain-like protein (MLKL), culminating in necroptosis and inflammation of the skin. At the same time, UVB-induced activation of RIPK3 also played a role in promoting the mitochondrial apoptotic pathway of Keratinocytes. In conclusion, UVB irradiation initiates an inflammatory response via RIPK1 pathway without necessitating its enzymatic activity. Simultaneously, RIPK3 can be activated by UVB exposure independently of RIPK1’s activity, resulting in necroptosis and inflammation of the skin.

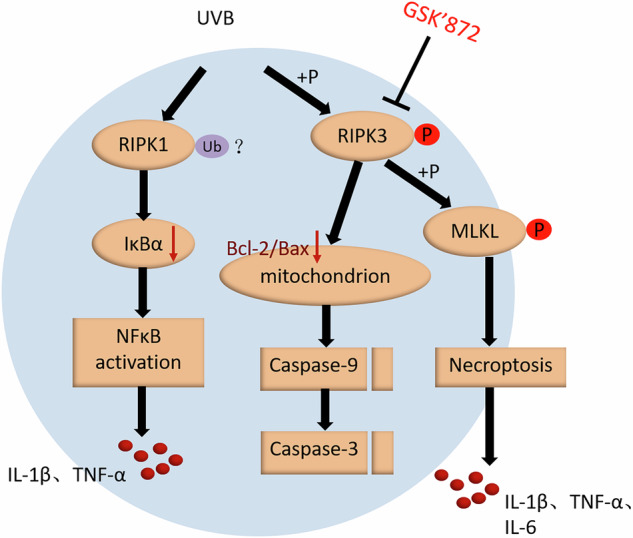

## Introduction

Ultraviolet A from sunlight penetrate deep into the dermis of human skin leading to damage of dermal collagens and skin photoaging. Conversely, ultraviolet B (UVB) is mostly absorbed by the epidermis and possess higher energy, making it more prone to inciting skin inflammation [1, 2]. Following UVB irradiation, keratinocytes generate photoproducts and oxidative byproducts, which induce a series of biological signal transductions. These transductions determine whether keratinocytes initiate damage repair programs to foster cellular inflammation or undergo apoptosis to become sunburned cells [3, 4]. When keratinocytes opt for the damage repair program, the activation of nuclear factor-kappa B (NF-κB) ensues. This, in turn, promotes the synthesis and release of inflammatory factors, including tumor necrosis factor (TNF)-α, interleukin (IL)-1β, and IL-6 [5, 6]. However, when UVB causes irreparable damage to keratinocytes, it triggers caspase activation and apoptosis of these cells [7]. This process serves to eliminate those cells prone to stable gene mutations, thereby reducing risks of malignancy.

Receptor-interacting protein kinase 1 (RIPK1) stands as a vital regulatory molecule in the context of cellular inflammation and necroptosis [8]. Activation of death receptors on the cell membrane triggers recruitment of RIPK1 by the death domain situated on inner side, initiating its ubiquitination. This ubiquitination, in turn, leads to phosphorylation and subsequent degradation of inhibitor of NF-κB (IκB), contributing to activation of NF-κB. Notably, NF-κB not only drives the transcription of pro-inflammatory genes but also induces the expression of pro-survival genes [9, 10]. Upon deubiquitination of RIPK1, its kinase activity is activated, subsequently recruiting and activating RIPK3 [11]. This activation sets the stage for the recruitment of the mixed-lineage kinase domain-like pseudo kinase (MLKL) to the RIPK1-RIPK3 complex and phosphorylated by RIPK3. The translocation of RIPK1-RIPK3-MLKL complex to the cell membrane serves as the catalyst for the execution of necroptosis [12, 13]. Unlike apoptosis, necroptosis also contributes to the generation of inflammatory factors and release of danger-associated molecular patterns (DAMPs), thus promoting inflammation [14]. Of note, researches have demonstrated that necrostatin-1 (Nec-1), a small molecule inhibitor of necroptosis, can effectively impede RIPK1 kinase activity and mitigate the associated inflammatory response [11]. Therefore, RIPK1 may be an important therapeutic target for some inflammatory diseases.

RIPK3 acts as a key regulatory molecule that not only activates MLKL to induce cell membrane permeabilization, but also affects mitochondria-associated metabolic enzymes, leading to respiratory burst and significant production of ROS [15, 16]. In contrast to apoptosis, RIPK3-mediated necroptosis leads to intracellular ATP depletion and the secretion of inflammatory factor IL-1β by promoting the activation of inflammasomes [17–19]. Consequently, it is evident that RIPK3 plays a crucial role not only in the initiation of necroptosis but also in driving a more severe inflammatory response.

In our earlier investigations, we identified the involvement of receptor-interacting proteins in UVB-induced photodamage of NIH3T3 cells. However, the precise regulatory mechanism remains incompletely elucidated [20]. As an important regulatory molecule within the NF-κB signaling pathway, RIPK1 holds the potential to play a significant role in UVB-induced inflammation in HaCaT cells. Existing research has demonstrated that ultraviolet radiation can prompt various forms of programmed cell death in keratinocytes, including apoptosis, pyroptosis, and autophagy [3, 21]. It is widely recognized that various cells, when subjected to physical, chemical damage or microbial infection, may exhibit characteristics associated with necroptosis [22]. Nevertheless, there remains a paucity of information regarding whether UVB-induced KC death involves RIPK1-mediated necroptosis. In addition, both RIPK1 and RIPK3 stand as crucial regulatory molecules in the process of cell necroptosis. Their protein expression levels and kinase activities have a pronounced impact on cell inflammation and cell death [21]. Among the RIPKs, the activity of RIPK3 is imperative for the initiation of necroptosis, while RIPK1-activity-independent mechanisms have emerged [11, 23]. Since the uncharted territory surrounding the roles and interactions of RIPK1 and RIPK3 in UVB radiation-induced damage to epidermis, this study endeavors to explore the mechanisms of these two molecules in UVB-induced keratinocytes death and inflammation of epidermis.

## Results

### RIPK1 promotes UVB-induced inflammation by activating NF-κB

**Fig. 1:**
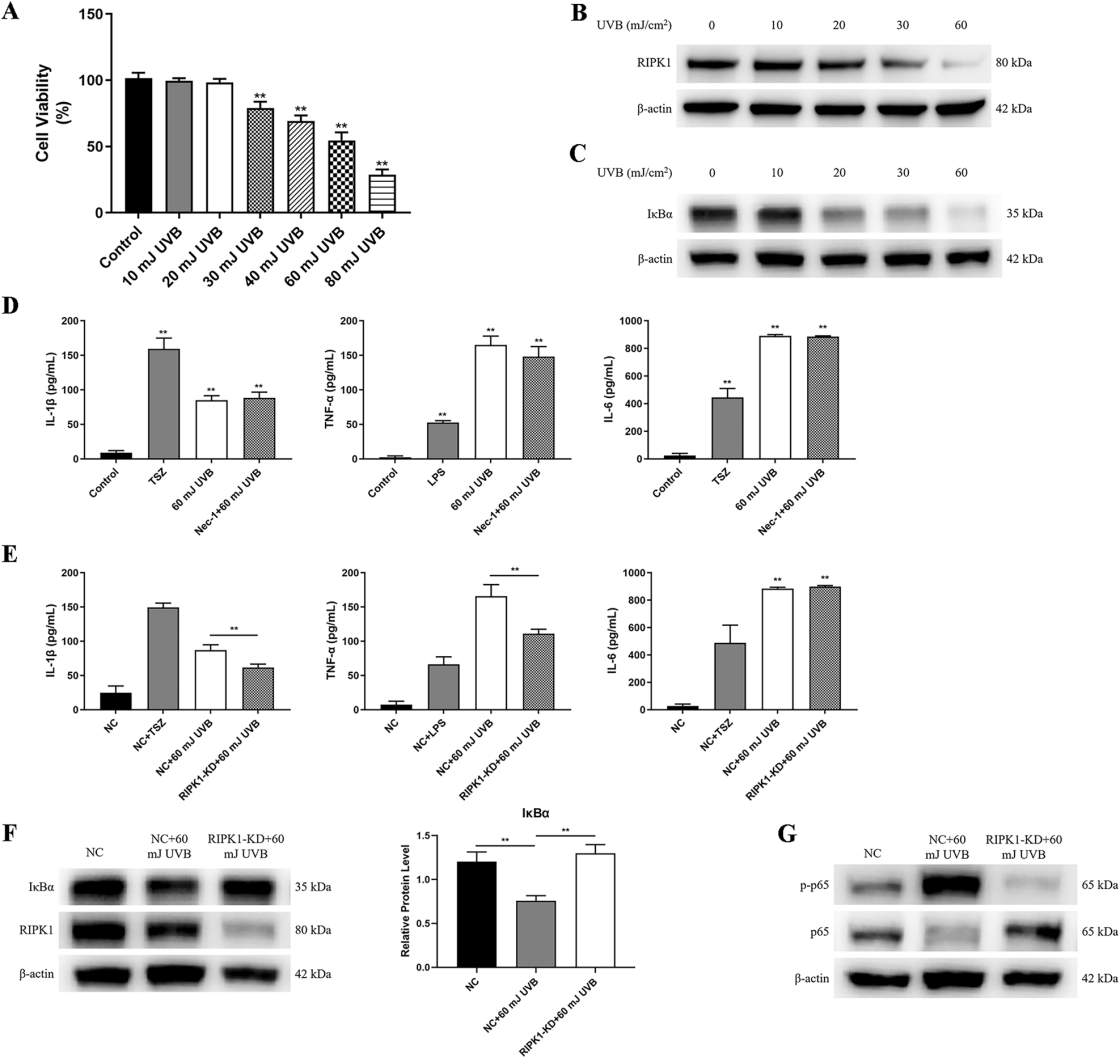
RIPK1 contributes to the cell inflammation after UVB irradiation by activating NF-κB. **A** Cell viability of HaCaT cells 24 h after UVB irradiation at varying doses assessed using the CCK-8 assay. Western blotting of RIPK1 (**B**) and IкBα (**C**) in HaCaT cells at 24 h after 60 mJ/cm^2^ UVB irradiation. **D** Levels of IL-1β, TNF-α, and IL-6 in the medium measured by ELISA 12 h after irradiation of HaCaT cells and 10 µM Nec-1-pretreated HaCaT cells with 60 mJ/cm² UVB; TSZ and LPS treatments serving as positive controls. **E** Levels of IL-1β, TNF-α, and IL-6 in the medium measured by ELISA 12 h after irradiation of NC HaCaT and RIPK1-KD HaCaT cells with 60 mJ/cm² UVB; TSZ and LPS treatments serving as positive controls. **F** Western blotting and statistical analysis of the relative protein level of IκBα in NC HaCaT and RIPK1-KD HaCT cells 12 h after 60 mJ/cm^2^ UVB irradiation. **G** Western blot analysis of phosphorylated (p)-p65 and total p65 in NC HaCaT and RIPK1-KD HaCT cells 12 h after 60 mJ/cm^2^ UVB irradiation. TSZ: 20 ng/mL TNF-α + 100 nM SM-164 + 20 μM Z-VAD(OMe)-FMK; LPS: 20 μg/mL LPS; NC negative control; RIPK1-KD: RIPK1-knockdown. Data are presented as mean ± SD, *n* = 3. *P* values determined by one-way analysis of variance (ANOVA), *P < 0.05, **P < 0.01.

In vitro cultured HaCaT cells were subjected to varying doses of UVB radiation, and the cell viabilities were assessed 24 h later by CCK-8 assay. It was observed that HaCaT cell viabilities began to exhibit significant declines at a UVB dose of 30 mJ/cm² compared to controls (Fig. 1A). Consequently, further experiments involved in irradiating HaCaT cells with UVB at doses of 10, 20, 30, and 60 mJ/cm². Western blot analysis revealed a reduction in the levels of RIPK1 proteins in the cell lysates as the doses of UVB irradiation increased. Specifically, UVB at 30 mJ/cm² and 60 mJ/cm² led to significant decreases in RIPK1 protein levels compared to the controls (Fig. 1B). As RIPK1 protein governs the degradation of IκBα in inflammatory pathways mediated by death receptors and pattern recognition receptors, we further investigated the alterations of IκBα at the protein levels following UVB exposure. Subsequent to UVB irradiation at doses of 10, 20, 30, and 60 mJ/cm², it was noted that the levels of IκBα protein also decreased in a dose-dependent manner. Notably, UVB irradiation at doses of 20, 30, and 60 mJ/cm² resulted in significant reductions in IκBα protein levels comparing with control group (Fig. 1C). These results suggest that UVB radiation exerts an influence on the protein levels of both RIPK1 and IκBα in HaCaT cells.

Nec-1, an inhibitor of RIPK1 kinase, functions to reduce cell necroptosis by suppressing the activity of RIPK1 kinase [11]. The viability of HaCaT cells treated with various concentrations of Nec-1 for 48 h was assessed, revealing that concentrations below 250 μM did not significantly affect cell viability (Fig. S1A). In addition, A concentration of 10 μM Nec-1 was employed to effectively block RIPK1 phosphorylation induced by TSZ (Fig. S1B). To investigate whether the kinase activity of RIPK1 influences UVB-induced inflammation in HaCaT cells, we treated the cells with 10 μM Nec-1 prior to UVB irradiation at 60 mJ/cm². As positive controls, we employed necroptotic HaCaT cells induced by 20 ng/mL TNF-α, 100 nM SM-164, and 20 μM Z-VAD(OMe)-FMK (referred to as TSZ), as well as TNF-α secretion stimulated by 20 μg/mL LPS. ELISA assays of the cell supernatants revealed significant increases of IL-1β, TNF-α, and IL-6 induced by UVB-irradiation, but these increases could not supressed by Nec-1 pretreatment (Fig. 1D). RIPK1 was knocked down in HaCaT cells using a lentiviral plasmid, resulting in RIPK1-KD HaCaT cells, while a blank plasmid served as the negative control to generate NC HaCaT cells. Both cell lines were exposed to 60 mJ/cm² UVB radiation. It was found that RIPK1-knockdown substantially inhibited the UVB-induced secretion of IL-1β and TNF-α although it did not affect IL-6 secretion (Fig. 1E). These findings suggest that the protein level of RIPK1, rather than its kinase activity, plays a role in UVB-induced inflammation in HaCaT cells, and that RIPK1 contributes to the secretion of inflammatory factors by UVB irradiation.

In order to investigate the regulation of the NF-κB inflammatory pathway by RIPK1 in UVB-induced damage to HaCaT cells, both NC HaCaT cells and RIPK1-KD HaCaT cells were subjected to 60 mJ/cm² UVB irradiation, with unirradiated NC HaCaT cells as control. Western blot analysis was employed to assess the levels of IκBα and phosphorylated NF-κB p65 (p-p65, the activated form of NF-κB). Notably, UVB irradiation led to a reduction in IκBα protein levels in HaCaT cells, while the knockdown of RIPK1 mitigated this reduction (Fig. 1F). In a corresponding manner, irradiation with 60 mJ/cm² UVB resulted in elevation of p-p65 levels in the cells, and this effect was alleviated by the knockdown of RIPK1 (Fig. 1G). These results suggest that RIPK1 promotes the production of UVB-induced inflammatory cytokines in HaCaT cells by down-regulating the protein levels of IκBα, thereby activating NF-κB.

### RIPK1 is not involved in UVB-induced cell death via kinase activity

**Fig. 2:**
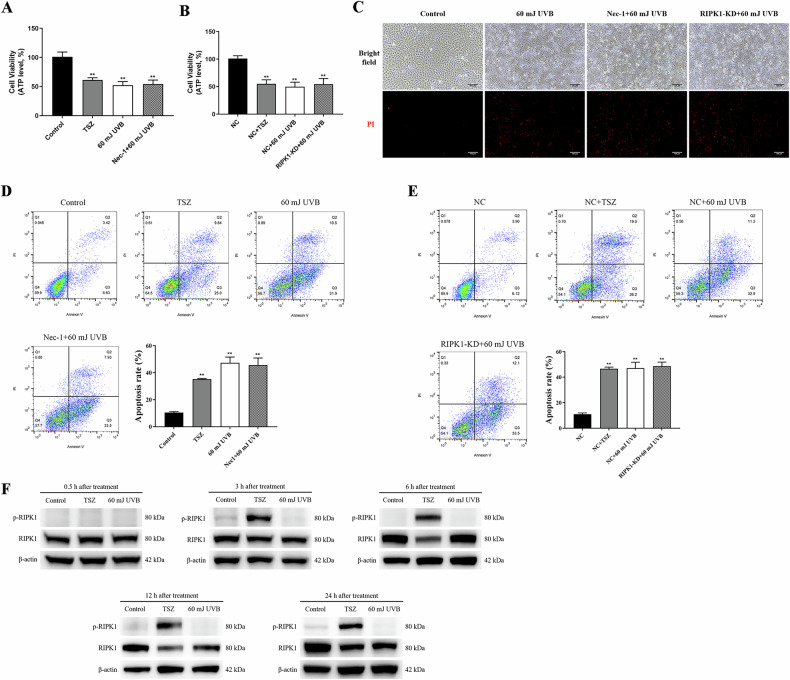
RIPK1 is not involved in UVB-induced cell death via kinase activity. **A** Cell viability of HaCaT cells and 10 µM Nec-1-pretreated HaCaT cells 24 h after 60 mJ/cm² UVB irradiation. **B** Cell viability of NC HaCaT cells and RIPK1-KD HaCaT cells 24 h after 60 mJ/cm² UVB irradiation. Viability in both (**A**) and (**B**) determined based on ATP levels; TSZ treatment serving as positive control. **C** PI fluorescent staining of HaCaT, Nec-1 pretreated HaCaT and RIPK1-KD HaCaT cells 24 h after 60 mJ/cm^2^ UVB irradiation; red staining indicating dead cells only (scale bar: 100 μm). **D** Apoptosis rates of HaCaT cells and Nec-1-pretreated HaCaT cells 24 h after 60 mJ/cm^2^ UVB irradiation. **E** Apoptosis rates of NC HaCaT and RIPK1-KD HaCaT cells 24 h after irradiated with 60 mJ/cm^2^ UVB. **F** Western blot analysis of phosphorylated (p)-RIPK1 and total RIPK1 in HaCaT cells at 0.5, 3, 6, 12, and 24 h following TSZ treatment or 60 mJ/cm² UVB irradiation. Data are presented as mean ± SD, *n* = 3. *P* values were determined by one-way analysis of variance (ANOVA), *P < 0.05, **P < 0.01.

The pivotal role of RIPK1 kinase activity in cell necroptosis, characterized by rapid ATP depletion, has been well established [24]. Therefore, we assessed HaCaT cell viability by measuring ATP levels after subjecting them to 60 mJ/cm² UVB irradiation or pretreatment with 10 μM Nec-1 prior to irradiation. It was observed that UVB irradiation significantly reduced cell viability, while Nec-1 had no impact on the decrease in cell survival rates following UVB irradiation (Fig. 2A). Similarly, when both NC HaCaT cells and RIPK1-KD HaCaT cells were exposed to 60 mJ/cm² UVB irradiation, the ATP levels remained relatively unchanged, indicating that UVB irradiation did not significantly affect the viability of RIPK1-KD HaCaT cells compared to NC HaCaT cells (Fig. 2B). Subsequently, HaCaT cells, Nec-1 pretreated HaCaT cells, and RIPK1-KD HaCaT cells were irradiated with 60 mJ/cm² UVB. 24 h later, the cells were stained with the red fluorescent dye propidium iodide (PI). Examination under fluorescence microscopy revealed that UVB irradiation resulted in substantial cell death, which was not ameliorated by either Nec-1 pretreatment or RIPK1 knockdown (Fig. 2C). In addition, the effects of varying concentrations of Nec-1 (10 μM, 50 μM, and 100 μM) on UVB-induced cell death were evaluated. Pretreatment with Nec-1 at all tested concentrations was found to be ineffective in preventing HaCaT cell death (Fig. S1C). Further analysis employing flow cytometry demonstrated that neither Nec-1 pretreatment nor RIPK1 knockdown significantly impacted the UVB-induced increase in apoptosis rates among the different HaCaT cell types (Fig. 2D, E). These findings indicate that neither the kinase activity of RIPK1 nor its protein levels are implicated in UVB-induced cell death in HaCaT cells.

Furthermore, HaCaT cells were collected at 0.5, 3, 6, 12, and 24 h following exposure to 60 mJ/cm² UVB irradiation, and the cell lysates were subjected to Western blot analysis. The results indicated that TSZ successfully induced RIPK1 phosphorylation from the 3rd hour following administration, whereas p-RIPK1 remained undetectable at all time points after UVB irradiation (see Fig. 2F). This suggests that while UVB irradiation can impact RIPK1 protein levels in HaCaT cells, it does not induce the activation of RIPK1 kinase.

### RIPK3 promotes UVB-induced cell death

**Fig. 3:**
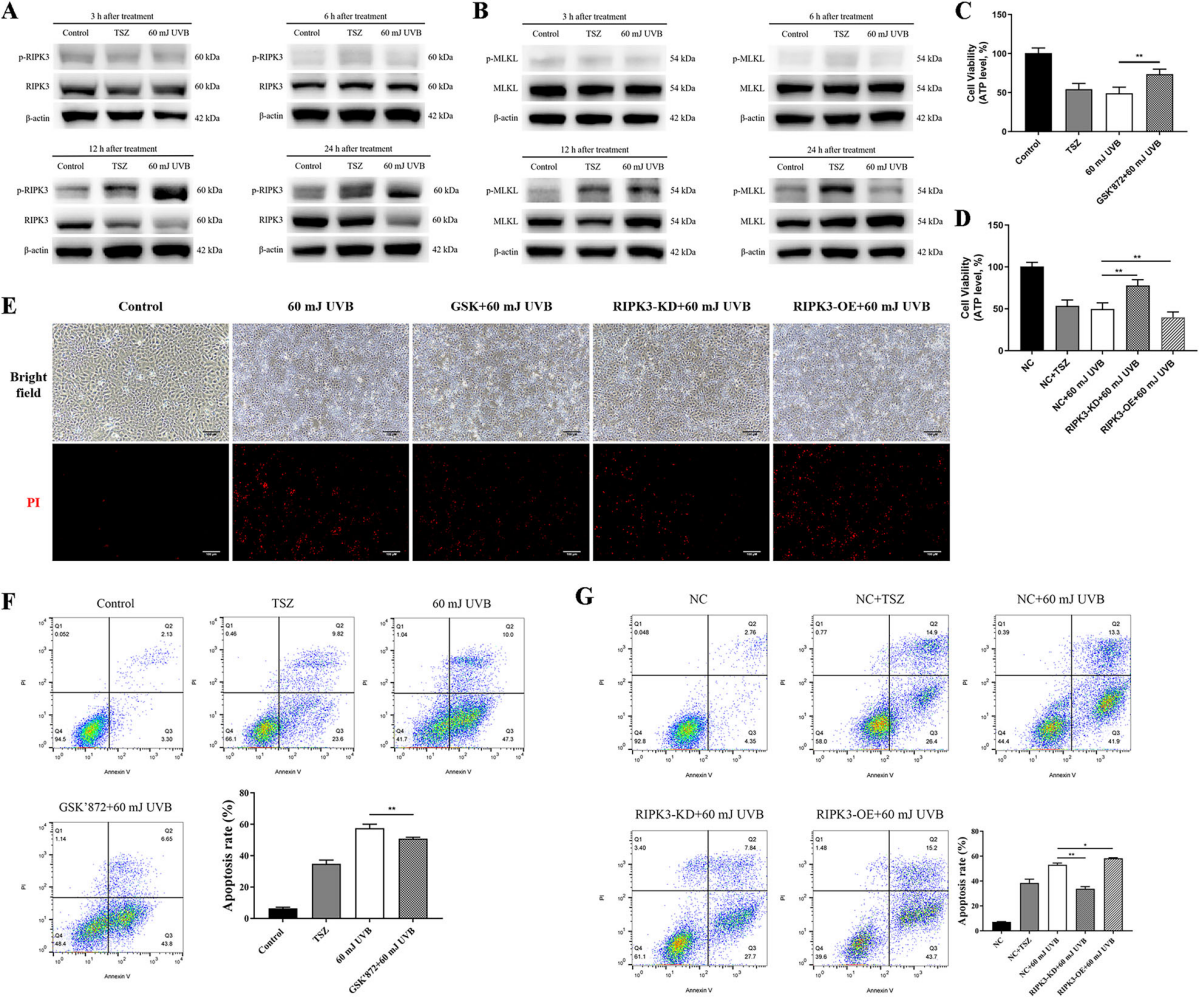
UVB-induced cell death is promoted by RIPK3. Western blotting of phosphorylated (p)-RIPK3, total RIPK3 (**A**), phosphorylated (p)-MLKL and total MLKL (**B**) in HaCaT cells at 3 h, 6 h, 12 h and 24 h following TSZ treatment or 60 mJ/cm^2^ UVB irradiation. **C** Cell viability of HaCaT and 10 µM GSK’872-pretreated HaCaT cells 24 h after 60 mJ/cm^2^ UVB irradiation. **D** Cell viability of NC HaCaT, RIPK3-KD HaCaT and RIPK3-OE cells 24 h after 60 mJ/cm^2^ UVB irradiation. Viability in both (**C**) and (**D**) determined based on ATP levels. **E** PI fluorescent staining of HaCaT, GSK’872 pretreated HaCaT, RIPK3-KD HaCaT and RIPK3-OE HaCaT cells at 24 h after 60 mJ/cm^2^ UVB irradiation; red staining indicating dead cells only (scale bar: 100 μm). **F** Apoptosis rates of HaCaT and GSK’872-pretreated HaCaT cells at 24 h after 60 mJ/cm^2^ UVB irradiation. **G** Apoptosis rates of NC HaCaT, RIPK3-KD HaCaT and RIPK3-OE HaCaT cells 24 h after 60 mJ/cm^2^ UVB irradiation. All TSZ treatment serving as positive control. RIPK3-KD: RIPK3 knockdown; RIPK3-OE: RIPK3 overexpression. Data are presented as mean ± SD, *n* = 3. *P* values determined by one-way analysis of variance (ANOVA), *P < 0.05, **P < 0.01.

Recent studies have reported that RIPK3 can be activated independently of RIPK1 kinase, particularly in response to microbial infections or specific environmental stresses [25, 26]. Therefore, it was hypothesized that the activation of RIPK3 kinase by UVB might induce cell death, even in the absence of observable effects on RIPK1 phosphorylation in HaCaT cells. In our subsequent experiments, HaCaT cells were exposed to 60 mJ/cm² UVB, and whole cell lysates were collected at 3, 6, 12, and 24 h after exposure. Notably, the levels of phosphorylated RIPK3 (p-RIPK3) and phosphorylated MLKL (p-MLKL) exhibited significant increases in the cells 12 h after UVB irradiation (Fig. 3A, B). Moreover, the elevated p-RIPK3 levels persisted for up to 24 h, while p-MLKL levels returned to baseline after 24 h (Fig. 3A, B). These findings suggest that RIPK3 continued to function as a kinase in HaCaT cells from 12 h after UVB irradiation. However, the activation of MLKL was not as sustained as that of RIPK3.

GSK’872 is a high-affinity RIPK3 kinase inhibitor that binds to its kinase domain, effectively reducing RIPK3-mediated necroptosis [27]. Cell viabilities, as assessed by the CCK-8 assay, revealed that the highest concentration of GSK’872 that did not adversely affect the viability of HaCaT cells was 50 μM (Fig. S2A). It was reported that the semi-effective concentration of GSK’872 for inhibiting RIPK3 kinase activity to be 1.3 nM [28]. Given that 10 μM GSK ‘872 was shown to effectively inhibit UVB-induced RIPK3 phosphorylation (Fig. S2B), this concentration was selected for subsequent experiments. The examination of cell viability revealed that UVB irradiation at 60 mJ/cm² led to a significant decrease in the survival rates of HaCaT cells. However, this effect was ameliorated by pretreatment with GSK’872 (see Fig. 3C). We subsequently generated RIPK3-knockdown HaCaT cells (designated as RIPK3-KD HaCaT cells) and RIPK3-overexpressing HaCaT cells (designated as RIPK3-OE HaCaT cells) using lentiviral plasmids. Following irradiation with 60 mJ/cm² UVB, RIPK3-KD HaCaT cells exhibited a rescue in cell survival rates compared to the UVB-exposed RIPK3-OE HaCaT cells (Fig. 3D). In contrast, the viabilities of RIPK3-OE HaCaT cells were significantly reduced when compared to NC HaCaT cells after UVB irradiation (Fig. 3D).

Moreover, NC HaCaT cells, GSK’872 pretreated NC HaCaT cells, RIPK3-KD HaCaT cells, and RIPK3-OE HaCaT cells were all subjected to PI staining 24 h after exposure to 60 mJ/cm² UVB irradiation. Examination under fluorescence microscopy revealed that although the extensive cell death induced by UVB exposure could be rescued by GSK'872 pretreatment or the absence of RIPK3, the overexpression of RIPK3 exacerbated cell death (Fig. 3E). Subsequent flow cytometry analysis showed a significant increase in apoptosis rates induced by 60 mJ/cm² UVB irradiation, which were similarly rescued by GSK’872 pretreatment or RIPK3 knockdown (Fig. 3F, G). Conversely, the overexpression of RIPK3 intensified cell apoptosis (Fig. 3G).

### RIPK3 mediates UVB-induced inflammation through necroptosis, independent of RIPK1 kinase activity

**Fig. 4:**
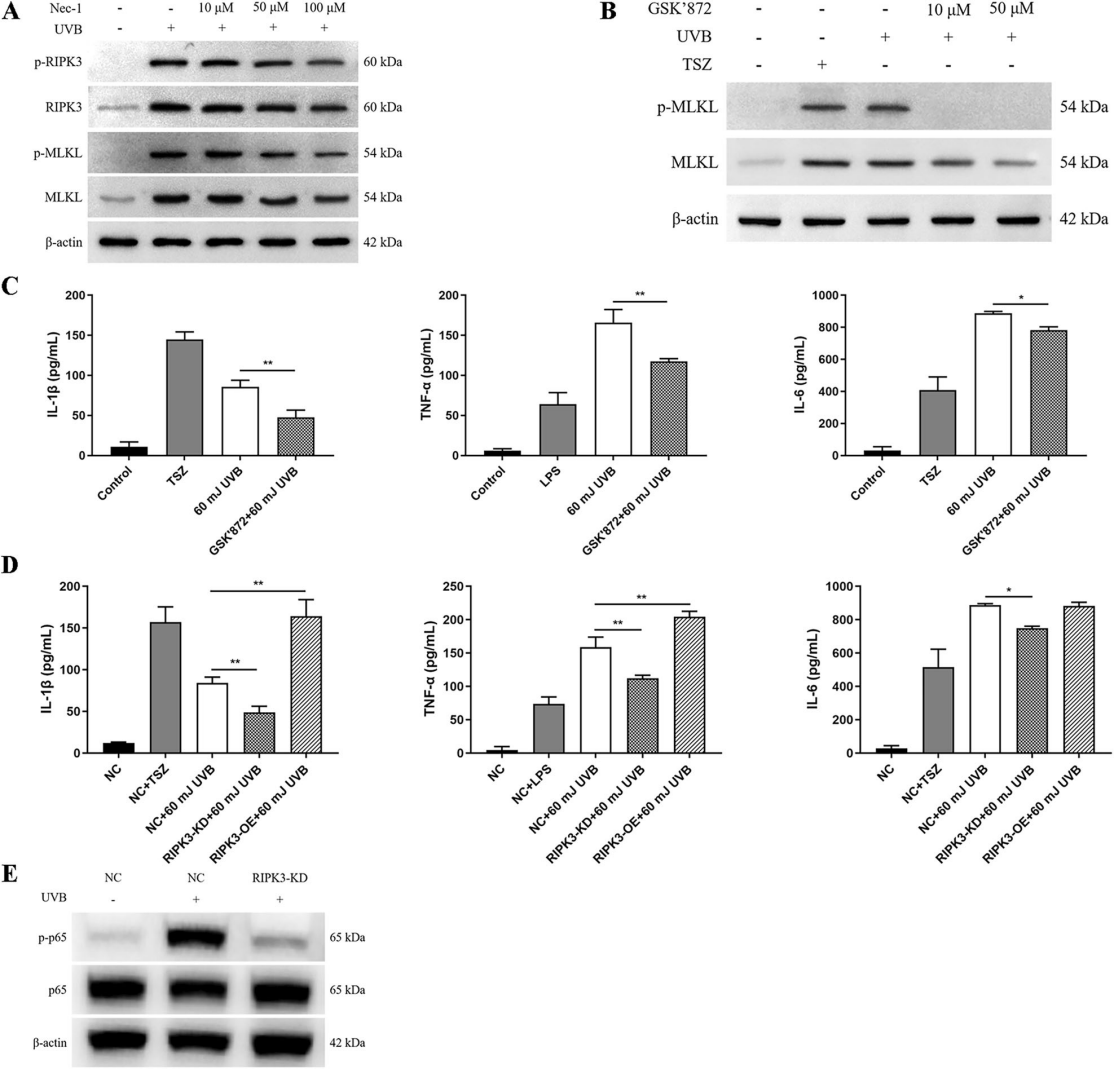
UVB-induced inflammation is mediated by RIPK3 through necroptosis, independent of RIPK1 kinase activity. **A** Western blot analysis of p-RIPK3, RIPK3, p-MLKL and MLKL in HaCaT cells pretreated with varying concentrations of Nec-1, assessed 12 h post-UVB irradiation. **B** Western blot analysis of p-MLKL and MLKL in HaCaT cells pretreated with 10 or 50 μM GSK’872, assessed 12 h post-UVB irradiation. **C** Levels of IL-1β, TNF-α and IL-6 in the medium detected by ELISA at 12 h after irradiation of HaCaT and GSK’872 pretreated HaCaT cells with 60 mJ/cm^2^ UVB. **D** Levels of IL-1β, TNF-α and IL-6 in the medium detected by ELISA at 12 h after irradiation of NC HaCaT, RIPK3-KD HaCaT and RIPK3-OE HaCaT cells with 60 mJ/cm^2^ UVB. **E** Western blot analysis of p-p65 and total p65 in NC HaCaT and RIPK3-KD HaCaT cells 12 h after 60 mJ/cm^2^ UVB irradiation. TSZ and LPS treatments serving as positive controls. Data are presented as mean ± SD, *n* = 3. P values determined by one-way analysis of variance (ANOVA), *P < 0.05, **P < 0.01.

To determine whether UVB-induced activation of RIPK3-MLKL is mediated by RIPK1 kinase activity, HaCaT cells were pretreated with 10, 50, or 100 μM Nec-1. Phosphorylation of RIPK3 and MLKL was assessed 12 h after UVB irradiation. The results showed that none of the tested Nec-1 concentrations significantly inhibited UVB-induced p-RIPK3 or p-MLKL (Fig. 4A). However, treatment with 10 or 50 μM RIPK3 inhibitor significantly reduced UVB-induced MLKL phosphorylation (Fig. 4B). These findings suggest that RIPK3 mediates UVB-induced necroptosis independently of RIPK1 kinase activity. To investigate whether UVB-induced activation of RIPK3 kinase exerts any influence on inflammation, HaCaT cells were pretreated with GSK’872 and irradiated with 60 mJ/cm² UVB. Cell supernatants were collected 12 h after the treatments for ELISA analysis. It was found that UVB exposure significantly stimulated the secretion of IL-1β, TNF-α, and IL-6 by HaCaT cells, while pretreatment with GSK’872 attenuated the levels of these cytokines (Fig. 4C). Following irradiation with 60 mJ/cm² UVB, it was observed that RIPK3 deficiency inhibited the UVB-induced secretion of IL-1β, TNF-α, and IL-6 in HaCaT cells, while the overexpression of RIPK3 enhanced the secretion of these cytokines (Fig. 4D). Additionally, the p-p65 level in RIPK3-KD HaCaT cells was significantly lower than that in NC HaCaT cells after exposure to 60 mJ/cm² UVB irradiation (Fig. 4E).

### RIPK3 contributes to the UVB-activated mitochondrial apoptotic pathway

**Fig. 5:**
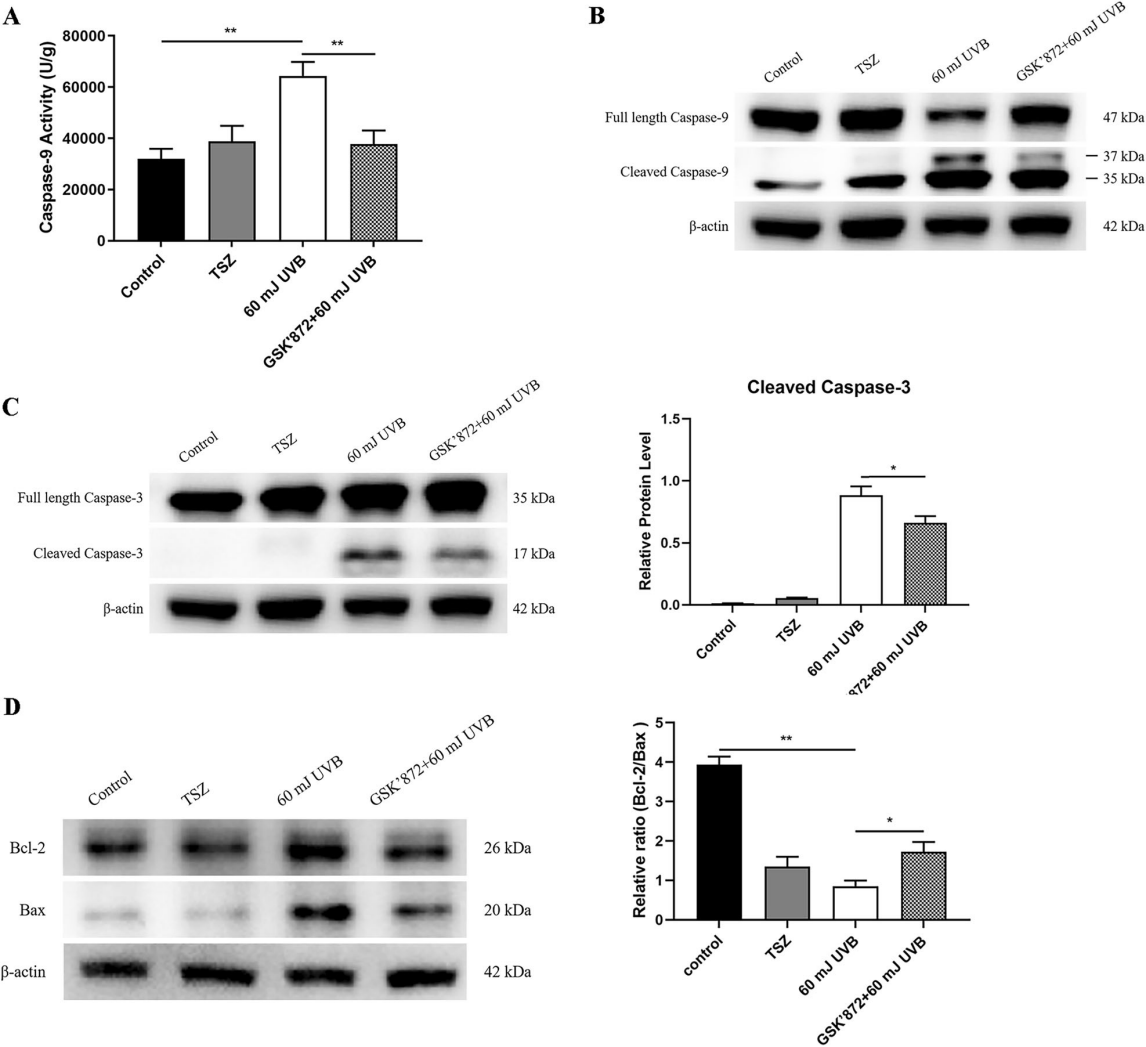
RIPK3 contributes to the activation of mitochondrial apoptotic pathway induced by UVB irradiation. **A** Caspase-9 activity of HaCaT and GSK’872 pretreated HaCaT cells at 24 h after 60 mJ/cm^2^ UVB irradiation. Western blot analysis of **B** cleaved caspase-9, full-length caspase-9, and **C** cleaved caspase-3, full-length caspase-3 in HaCaT and GSK’872 pretreated HaCaT cells at 24 h after 60 mJ/cm^2^ UVB irradiation; and statistical analysis of the relative protein level of cleaved caspase-3. **D** Western blot analysis of bcl-2 and bax in HaCaT and GSK’872 pretreated HaCaT cells at 24 h after 60 mJ/cm^2^ UVB irradiation; and statistical analysis of the relative ratio of bcl-2/bax. Data are presented as mean ± SD, *n* = 3. *P* values determined by one-way analysis of variance (ANOVA), *P < 0.05, **P < 0.01.

It is widely recognized that different forms of programmed cell death exhibit interactions with one another [29, 30]. To investigate whether RIPK3 exerts an impact on the mitochondrial apoptotic pathway in addition to necroptosis, HaCaT cells were pretreated with 10 μM GSK’872 followed by 60 mJ/cm² UVB exposure. The experiments demonstrated a significant increase in caspase-9 activity in HaCaT cells after UVB irradiation, an effect that was notably suppressed by GSK’872 (Fig. 5A). Further analysis revealed that UVB irradiation induced a significant increase in the protein levels of cleaved caspase-9 and cleaved caspase-3 in HaCaT cells. Although cleaved caspase-9 p35 level did not significantly change with RIPK3 inhibition, cleaved caspase-9 p37 and cleaved caspase-3 were markedly downregulated (Fig. 5B, C). It is well established that full-length caspase-9 and caspase-3 must undergo cleavage into smaller fragments to become enzymatically active, the results therefore, indicated that the kinase activity of RIPK3 promoted the activation of caspase-9 and caspase-3 after UVB irradiation.

Furthermore, both Bcl-2 and Bax play important roles in mitochondrial apoptosis, where Bcl-2 functions as an inhibitor of apoptosis and Bax acts as a promoter. The relative ratio of Bcl-2 to Bax proteins can serve as an indicator of the activation of mitochondrial apoptotic pathway within cells [31]. A higher ratio signifies increased resistance of cells to apoptosis. In our experiments, both HaCaT cells and HaCaT cells pretreated with 10 μM GSK’872 were exposed to 60 mJ/cm² UVB irradiation. Western blot analysis revealed that UVB irradiation significantly reduced the relative ratio of Bcl-2 to Bax proteins, while the inhibition of RIPK3 kinase activity significantly increased this ratio in HaCaT cells (Fig. 5D). To assess the impact of apoptosis on RIPK3, HaCaT cells were treated with the pan-caspase inhibitor Z-VAD(OMe)-FMK (20 μM). Inhibition of the mitochondrial apoptotic pathway did not prevent UVB-induced RIPK3 phosphorylation (Fig. S3). These findings suggest that the mitochondrial apoptosis pathway induced by UVB is regulated by RIPK3 and occurs downstream of it.

### RIPK3 regulates UVB-induced epidermal necroptosis in mice

**Fig. 6:**
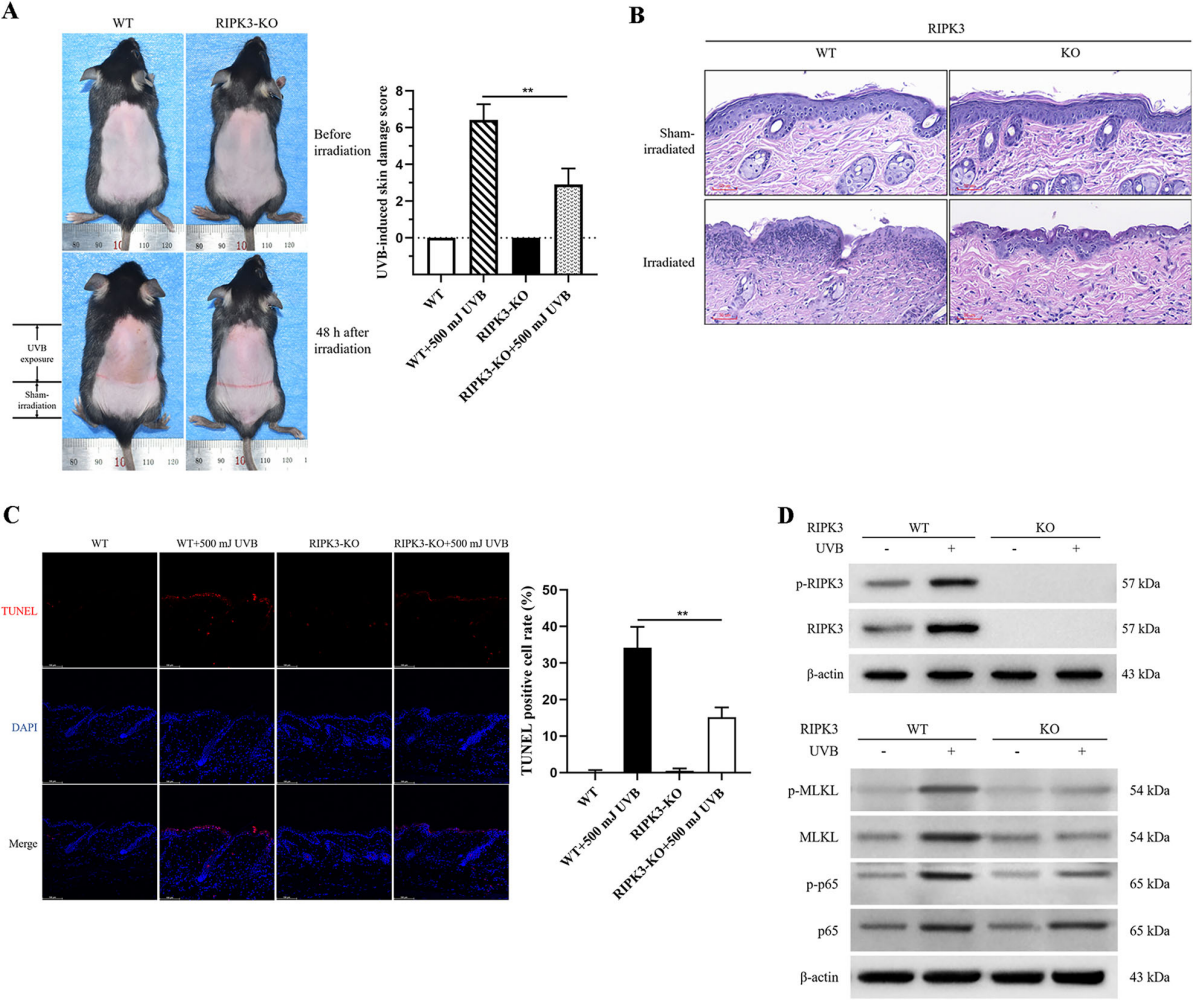
RIPK3 promotes UVB-induced epidermal necroptosis in mice. **A** Evaluation of dorsal skin lesions in WT (C57BL/6 J) and RIPK3-KO mice 48 h after UVB irradiation (500 mJ/cm²) or sham treatment, using a 12-point scoring system outlined in Table 1 (*n* = 6). **B** Histopathological analysis of dorsal skin from (**A**) using H&E staining, with representative images selected to illustrate key features (scale bar: 100 μm). **C** TUNEL assay of dorsal skin from mice in (**A**), providing representative images of epidermal cell apoptosis and quantification of apoptosis rate based on TUNEL-positive cells (*n* = 5). **D** Western blot analysis of p-RIPK3, p-MLKL, and p-NFκB in skin samples from (**A**) (*n* = 3). Data are presented as mean ± SD. *P* values determined by one-way analysis of variance (ANOVA), *P < 0.05, **P < 0.01.

To ascertain the involvement of RIPK3 in UVB-induced skin damage, mice of the WT C57BL/6 J strain were subjected to varying doses of UVB irradiation. Pronounced inflammatory skin manifestations including erythema, edema, blister, erosion and ulceration, manifested at 48 h post-500 mJ UVB exposure (Fig. 6A). Histopathological examination and TUNEL staining revealed extensive necrosis and apoptosis of keratinocytes (Fig. 6B, C). Concurrently, there was a notable increase in the levels of necroptosis-associated molecules, namely p-RIPK3 and p-MLKL proteins, as well as the pro-inflammatory factor p-NFκB (Fig. 6D).

Moreover, UVB irradiation at a dose of 500 mJ on the dorsal skin of RIPK3 knockout (RIPK3-KO) mice demonstrated a significant mitigation of UVB-induced skin damage, as evidenced by decreased scores of skin damage (Table 1, Fig. 6A), as well as ameliorated necrosis and apoptosis of keratinocytes in the mouse epidermis (Fig. 6B, C). Notably, RIPK3 deficiency not only suppressed the elevation of p-MLKL levels induced by UVB, but also diminished the levels of the pro-inflammatory factor p-p65 in the epidermis of the mice (Fig. 6D).

**Table 1:** The severity of UVB-induced skin damage score scale.

Erythema	Edema and blister	Erosion, crust and ulceration	Score
None	None	None	0
Pink	Slight swelling, dermatoglyph exist	Erosion or crust, without exudation	1
Red	Obvious swelling, dermatoglyph disappear	Erosion or crust, with exudation	2
Dark red	Tense and shiny, or with blisters	Ulceration	3

## Discussion

RIPK1 regulates cellular inflammation and death through various post-translational modifications. Notably, ubiquitylated RIPK1 induces cellular inflammation and promotes cell survival by activating the NF-κB signaling pathway, whereas the kinase activity of RIPK1 triggers cell necroptosis or apoptosis [32, 33]. Our investigations have revealed that as the intensity of UVB irradiation increased, the expression levels of both RIPK1 and IκBα proteins gradually declined in HaCaT cells, which infers that UVB activates NF-κB by modulating RIPK1 protein. It is worth noting that toll-like receptors (TLRs) are known to become activated, thereby promoting the activation of the NF-κB signaling pathway when cells are exposed to UVB. Such activation leads to the synthesis of inflammatory cytokines such as IL-1β, TNF-α, IL-6, among others [4, 34–37]. Our results also demonstrated an increase in the levels of IL-1β, TNF-α, and IL-6 in the supernatants of HaCaT cells following irradiation with 60 mJ/cm² UVB. However, the levels of these inflammatory factors, which were largely unaffected by RIPK1 kinase inhibition, were reversed following RIPK1 knockdown. These results suggest that UVB-induced inflammation may not be driven by RIPK1 activation but rather regulated by changes in RIPK1 protein levels.

Furthermore, high-intensity (60 mJ/cm²) UVB irradiation significantly reduced the levels of IκBα protein while increasing the p-p65 protein within the cells. These effects could be rescued by the knockdown of RIPK1. It’s important to note that RIPK1 undergoes dynamic modifications through a complex ubiquitination pattern [33, 38]. Ubiquitination of RIPK1 leads to the activation of the NF-κB pathway and the production of inflammatory factors, while RIPK1 kinase activation results from its deubiquitination, promoting necroptosis in the cells [39, 40]. Therefore, the activation of NF-κB signaling pathway induced by UVB irradiation in HaCaT cells may be linked to RIPK1-mediated ubiquitination and degradation of IκBα. Additionally, our experiments demonstrated that UVB irradiation did not result in a significant increase in RIPK1 phosphorylation in HaCaT cells. Moreover, inhibition of RIPK1 kinase activity by various concentrations of Nec-1 did not mitigate UVB-induced cell death. The involvement of autocrine TNF in the regulation of cell death via TNFR in HaCaT cells following UVB irradiation has been previously suggested [6, 41]. However, earlier studies indicate that DNA damage can induce ubiquitin modification of RIPK1 independently of TNFR, which subsequently activates the downstream NF-κB signaling pathway [42, 43]. Consistent with our findings, it can be concluded that UVB irradiation, as a DNA damage inducer, primarily mediates keratinocyte inflammation rather than necroptosis through RIPK1 in the early stages of photodamage in HaCaT cells.

In this study, enhanced phosphorylation of RIPK3 and MLKL was observed in both HaCaT cells and mouse dorsal skin following UVB irradiation, signifying the induction of RIPK3 and MLKL activation in epidermis. Cell death induced by UVB irradiation could be rescued by inhibiting RIPK3 kinase with GSK'872 or by having RIPK3 deficiency. These findings strongly indicate that RIPK3 regulates UVB-induced keratinocyte death. It’s worth noting that various cellular stressors can trigger necroptosis through signaling pathways initiated by death receptors and pattern recognition receptors, such as TNFR1/TNF, Fas/FasL, TRAILR/TRAIL, TLR4/LPS [22]. RIPK3 plays a vital role in necroptosis, irrespective of the upstream triggering factors, serving as a key regulator of both cellular necroptosis and inflammatory responses. Recent studies have reported that RIPK1 kinase activity is not universally required for all necroptosis instances, as RIPK3 can be activated independently of RIPK1 by external stimuli, such as viral infection, heatstroke, and osmotic stress, which activate RIPK3 via Z-DNA-binding protein 1 (ZBP1) [23, 26, 44, 45]. Moreover, The UVB-induced RNA damage and ROS production can be detected by various receptors, including TLR3, NOD-like receptor pyrin domain-containing protein 1 (NLRP1), and NLRP3 [4, 46–48]. These receptors may mediate RIPK3 activation independently of RIPK1 kinase activity. Hence, it can be speculated that UVB-induced cell damage, likely through the activation of PRRs or ZBP1, directly activates RIPK3 in epidermal keratinocytes, leading to necroptosis.

To investigate the role of RIPK3 in UVB-induced necroptosis and inflammation, the effects of RIPK1 and RIPK3 kinase inhibitors on UVB-induced elevation of p-MLKL, a key executor of necroptosis, were examined. The results revealed that the UVB-induced increase in p-MLKL levels was inhibited by the RIPK3 inhibitor, but not by the RIPK1 inhibitor. In addition, the enhanced secretion of cytokines, including IL-1β, TNF-α, and IL-6 induced by 60 mJ/cm² UVB irradiation, was significantly dampened by the presence of GSK’872 and RIPK3 deficiency. Conversely, the overexpression of RIPK3 exacerbated the UVB-induced cytokine secretion, underscoring RIPK3’s pro-inflammatory role in HaCaT cells following UVB irradiation. It was observed that the deficiency of RIPK3 also restrained the elevation of p-p65 levels in both keratinocytes and epidermis after UVB irradiation. Previous studies have reported that RIPK3 not only triggers cell necroptosis and the release of inflammatory factors, such as IL-1β, but also enhances IL-1β secretion through the activation of NLRP3 [49, 50]. Although RIPK1 is known to be involved in promoting the production of inflammatory factors, our study demonstrates that RIPK3 regulates UVB-induced necroptosis independently of RIPK1 kinase activity, thereby amplifying the inflammatory response in the epidermis following UVB exposure.

It has been reported that UVB exposure can induce various forms of cell death, including apoptosis, pyroptosis, autophagy, and other mechanisms in keratinocytes. Specifically, caspase-9 acts as an upstream mediator in the mitochondrial apoptosis pathway triggered by UVB irradiation in human keratinocytes [51, 52]. It is activated by being cleaved into two subunits of different molecular weights, namely p37 and p35. p35 directly activates downstream caspase-3 [53], while p37 amplifies the caspase cascade to promote apoptosis [54]. UVB exposure results in increased levels of cleaved caspase-9 p37 and cleaved caspase-3 proteins in HaCaT cells, and the application of RIPK3 kinase inhibitors downregulates these levels. However, inhibition of caspases does not affect the phosphorylation of RIPK3. These results indicate that the mitochondrial apoptosis pathway is downstream of RIPK3 and is regulated by it. More importantly, the deficiency of RIPK3 effectively rescued the apoptosis observed in keratinocytes within the dorsal skin of UVB-irradiated mice. Notably, it is widely recognized that exposure to UVB activates the mitochondrial membrane of keratinocytes through Bax, thereby creating a channel for the release of apoptotic factors like cytochrome C [55]. In contrast, Bcl-2 plays a protective role against UVB-induced apoptosis by inhibiting mitochondrial outer membrane permeability [52]. Our study also reveals that UVB irradiation reduces the Bcl-2/Bax protein ratio in HaCaT cells, but this reduction can be counteracted by the RIPK3 kinase inhibitor. The relative Bcl-2/Bax protein ratio serves as an indicator of cell resistance to apoptosis, with a higher ratio signifying a stronger anti-apoptotic capability, and a lower ratio suggesting an increased propensity for apoptosis. The aforementioned observations suggest an additional role for RIPK3 in the modulation of epidermal keratinocytes apoptosis subsequent to UVB exposure. This modulation is executed through the facilitation of the activation of the mitochondrial apoptotic pathway within keratinocytes.

In summary, UVB irradiation induces RIPK1-mediated degradation of IκB in HaCaT cells, leading to the activation of the NF-κB pathway and subsequent production of inflammatory factors. Simultaneously, UVB radiation activates RIPK3 independently of RIPK1 activity, initiating the phosphorylation of downstream MLKL. This cascade ultimately induces necroptosis and augments the secretion of inflammatory cytokines in epidermal keratinocytes. RIPK3 also plays a role in the activation of mitochondrial apoptotic pathway in HaCaT cells induced by UVB. It is postulated that RIPK3 serve as a central player in orchestrating various forms of programmed cell death in keratinocytes simultaneously induced by UVB irradiation. Notably, GSK’872, a RIPK3 kinase inhibitor, demonstrates potential in rescuing UVB-induced cell death and inflammation, suggesting its relevance in the prevention and treatment of photodamage. Further experiments are warranted to confirm whether UVB irradiation leads to the ubiquitination of RIPK1 in HaCaT cells and how UVB activates RIPK3 kinase, potentially through pattern recognition receptors or Z-DNA-binding protein 1, among other molecules.

## Materials and methods

### Cell culture and UVB irradiation

The HaCaT keratinocyte cell line, sourced from the American Type Culture Collection (ATCC, Manassas, VA, USA), was cultured under standard conditions in Dulbecco’s modified Eagle’s medium (DMEM; Gibco) supplemented with 10% fetal bovine serum (FBS; HyClone, USA) and 1% antibiotics (100 U/mL penicillin and 100 µg/mL streptomycin). Cells were maintained in a humidified atmosphere containing 5% CO_2_ at 37 °C, with periodic authentication performed through short tandem repeat (STR) profiling to confirm cellular identity and absence of contamination. For subsequent experiments, cells were detached using 0.25% trypsin (HyClone, USA) and then seeded onto either cell culture plates or dishes. Upon reaching approximately 60% confluency, the culture medium was replaced with a solution containing either 10 μM Nec-1, 10 μM GSK’872 or 20 μM Z-VAD(OMe)-FMK (MedChemExpress, USA). Following this, the cells were further cultured for 24 h. Prior to UVB irradiation, the culture medium was aspirated, and the cells were washed twice with pre-warmed PBS buffer. Subsequently, the HaCaT cells were exposed to a Narrow Band-UVB therapeutic instrument (208T, Waldmann, Germany) for irradiation, with the UVB irradiation intensity being measured using a Waldmann UV Meter. Meanwhile, HaCaT cells treated with TSZ (20 ng/mL TNF-α, 100 nM SM-164, and 20 μM Z-VAD(OMe)-FMK) were used as a positive control for necroptosis. In the in vitro experiments, each experimental group comprised three independent biological replicates (*n* = 3). Sample allocation was randomized using a block design.

### Stable transfected HaCaT cell lines

Stable Negative control (NC), RIPK1-knockdown (RIPK1-KD), RIPK3-knockdown (RIPK3-KD), and RIPK3-overexpressing (RIPK3-OE) HaCaT cell lines were generated by infecting cells with recombinant lentivirus expressing control shRNA, RIPK1 shRNA, RIPK3 shRNA, and full-length RIPK3 respectively. The infection was performed at a multiplicity of infection (MOI) of 100 in a solution containing 5 μg/mL polybrene. Cells at 30–40% confluence were selected using 2.5 μg/mL puromycin dihydrochloride (Beyotime, Shanghai) for 14 days. The methods for lentivirus packaging are detailed in the supplementary materials.

### ELISA

Human IL-1β, TNF-α, and IL-6 ELISA kits (Boster; Wuhan) were utilized to detect the inflammatory factors and cytokines in the supernatants of both normal and transfected HaCaT cells. Briefly, following various treatments, the culture media of the cells were harvested and spun for 5 min at 1000 × *g* Supernatants were collected and diluted and subsequently added to the microplates that had been precoated with antibodies specific to IL-1β, TNF-α, or IL-6, with each well receiving 100 μL of the solution. After incubation for 90 min at 37 °C in a constant-temperature incubator, an automatic plate washer was employed to remove the liquid from the microplates. Subsequently, incubations with diluted biotin-conjugated anti-human IL-1β, TNF-α, or IL-6 antibodies for 1 h at 37 °C were followed by reactions with avidin-peroxidase complex working solutions for 30 min at 37 °C. This step was succeeded by incubation with 90 μL of TMB chromogenic solution in each well for 20–25 min at 37 °C in the dark. Finally, a termination solution was introduced at 100 μL per well, and the optical density (O.D.) values at 450 nm were measured using a microplate reader (EnSpire, PerkinElmer, USA).

### Cell viability

Normal and stable transfected HaCaT cells were plated in 96-well opaque plates with a black background, while wells containing medium without cells served as controls. Cell viability was assessed using the CellTiter-Glo® Luminescent Cell Viability Assay kit (Promega, USA) following various treatments. Upon addition of an equivalent volume of CellTiter-Glo® Reagent to the cell culture medium in each well, the contents were mixed on an orbital shaker for 2 min, leading to cell lysis. Luminescence signals were quantified employing a microplate reader (EnSpire, PerkinElmer, USA) after an incubation period of 10 min at room temperature.

### PI staining

Cells were seeded into 12-well plates and subjected to various treatments. Following treatment completion, the cells were gently rinsed with PBS and subsequently exposed to a 50 μg/mL propidium iodide (PI) staining solution (Beyotime, Shanghai) for a duration of 10 min, all under room-temperature conditions and in darkness. Following two rounds of PBS washing, the cells were scrutinized and photographed using a fluorescence microscope (Leica, Germany).

### Flow cytometry

Cells were planted in 12-well plates and went through individualized treatment protocols. Apoptosis was assessed utilizing Annexin V-FITC apoptosis detection kits (Beyotime, Shanghai). Briefly, cells were detached with 0.25% trypsin, followed by centrifugation at 1000 × *g* for 5 min. After removal of the supernatant, cells were resuspended in PBS. A total of 1 × 10^5^ resuspended cells underwent centrifugation at 1000 × *g* for 5 min to eliminate the supernatant and resuspended gently with 195 μl of Annexin V-FITC binding solution. Following the admixture with 5 μL of Annexin V-FITC and 10 μL of PI staining solution, the cells were incubated at room temperature in darkness for 10–20 min. Subsequently, they were analyzed using a flow cytometer (BD Biosciences, New Jersey).

### Caspase activity

Cells were plated into 12-well dishes, and the activity of caspase-9 was assessed using a commercially acquired caspase-9 activity assay kit (Beyotime, Shanghai). Briefly, cells were detached with trypsin and gathered into centrifuge tubes, followed by centrifugation at 600 × *g* to collect the cells. The supernatants were retrieved post-centrifugation of fully lysed cells at 20,000 × *g* and maintained at 4 °C. The reaction system was configured as follows: 40 μL of detection buffer, 50 μL of the sample’s supernatant to be tested, and 10 μL of Ac-LEHD-pNA (2 mM). The reagents were evenly mixed with each sample in a 96-well plate according to the reaction system, and a blank control, containing lysate without a sample, was employed. Following incubation at 37 °C for 120 min, O.D. values were measured at 405 nm utilizing a microplate reader (EnSpire, PerkinElmer, USA) after color development, with subsequent calculation of enzyme activity units in each sample. Concurrently, a small portion of the samples was taken to detect protein concentration via the bradford protein concentration assay kit (Beyotime, Shanghai). The ratio of enzyme activity units to protein concentration was referred to as the enzyme activity unit of caspase-9 per gram of sample proteins.

### Animal models and UVB radiation

Wild-type (WT) C57BL/6J mice were purchased from Medical Discovery Leader (Beijing, China). The RIPK3-knockout (RIPK3-KO) mice, generously provided by Professors Jiahuai Han at Xiamen University and Ben Lu at Central South University [23], were identified by PCR genotyping. Mice were raised in specific pathogen-free grade animal laboratories with unrestricted access to food and water. All experimental procedures involving animals were reviewed and approved by the Review Board of Plastic Surgery Hospital, Chinese Academy of Medical Sciences (approval number EAEC 2023-012#), in accordance with established ethical guidelines for biomedical research. In our study, 8-week-old female mice of either WT or RIPK3-KO genotype weighing approximately 18–22 g, were randomly allocated to respective experimental groups using a block design (*n* = 6/group). Before UVB exposure, mice were anesthetized, and their dorsal hairs were removed. Subsequently, a black shade was applied to the caudal side of the dorsal skin (sham-irradiation). UVB irradiation was administered using a UVB therapeutic instrument at a total dose of 500 mJ/cm^2^. At 48 h post irradiation, the mice were euthanized for photographic documentation, assessment of skin injuries, and harvesting of skin samples for subsequent experiments.

### Assessment of UVB-induced skin damage severity

The assessment of UVB-induced skin damage severity employed a 12-point scale (Table 1). Distinct categories of skin lesions were assigned scores based on their respective severity levels: 0 for none, 1 for mild, 2 for moderate, and 3 for severe. Quantitative assessment of UVB-induced cutaneous damage was conducted by two dermatologists under double-blinded condition. Prior to evaluation, each mouse was assigned a unique identification code to ensure allocation concealment. Inter-rater discrepancies resolved through consensus discussion and re-examination of photographic documentation.

### Tissue histopathology

Following fixation of murine dorsal skin tissues in 4% paraformaldehyde, the specimens underwent paraffin embedding. Paraffin sections were subsequently prepared for hematoxylin-eosin (H&E) staining. Observations and photomicrography were performed utilizing a light field camera (Motic, Xiamen) while examining the sections under a microscope.

### TUNEL apoptosis assay

Following deparaffinization of the sections in xylene, hydration was achieved through exposure to varying concentrations of ethanol. Subsequently, sections were treated with 20 μg/mL DNAase-free proteinase K (Beyotime, Shanghai) at 37 °C for 30 min. After washing with PBS, tissue staining was performed using the One Step TUNEL Apoptosis Assay Kit (Beyotime, Shanghai). Observations and photomicrographs were obtained utilizing a confocal microscope (Leica, Germany).

### Western blot

HaCaT cells were lysed using SDS lysis buffer (Beyotime, Shanghai), followed by low-temperature centrifugation to obtain total proteins. Mouse skin tissues were cut into small fragments, and SDS lysis buffer was added at a volume ten times that of the tissue. The tissue was then homogenized using a homogenizer, and the supernatant was collected to obtain the total protein solution. After that, the proteins were separated using 4–12% gradient sodium dodecyl sulfate-polyacrylamide gel electrophoresis (SDS-PAGE) at 30 μg per lane. The proteins within the gel were transferred onto a polyvinylidene fluoride (PVDF) membrane using electrophoretic transfer. After blocking, the membrane was incubated with primary antibodies that were appropriately diluted and maintained at 4 °C for 12–16 h. The PVDF membranes were then washed and incubated with a horseradish peroxidase-conjugated secondary antibody (SA00001-1, SA00001-2; Proteintech) for 1 h at room temperature. β-actin was used as loading control in these experiments. Finally, the target protein bands on the PVDF membrane were visualized using an enhanced chemiluminescence detection kit (Applygen, Beijing) on a chemiluminescence instrument (BIO-RAD, USA). The intensity of the target protein bands was statistically analyzed with Image J software. Western blotting analyses were performed using the following antibodies: Anti-Phospho-RIP (Ser166) (#65746, Cell Signaling Technology), Anti-RIP (#3493, Cell Signaling Technology), Anti-IκB alpha (ab32518, abcam), Anti-NF-kB p65 (phospho S536) (ab76302, abcam), Anti-NF-κB p65 (#8242, Cell Signaling Technology), Anti- Phospho-RIP3 (Ser227) (#93654, Cell Signaling Technology), Phospho-RIP3 (Thr231/Ser232) (#91702, Cell Signaling Technology), Anti-RIP3 (#13526, Cell Signaling Technology), Anti-RIP3 (#95702, Cell Signaling Technology), Anti-Phospho-MLKL (Ser358) (#91689, Cell Signaling Technology), Anti-Phospho-MLKL (Ser345) (#37333, Cell Signaling Technology), Anti-MLKL (ab184718, abcam), Anti-MLKL (#26539, Cell Signaling Technology), Anti-Caspase-9 (#9502, Cell Signaling Technology), Anti-Caspase-3 (#9662, Cell Signaling Technology), Anti-Bcl-2 (#4223, Cell Signaling Technology), Anti-Bax (#2772, Cell Signaling Technology), Anti-β-Actin (#5125, Cell Signaling Technology).

### Statistical analysis

Statistical analyses were performed using *GraphPad Prism* 7.0 (GraphPad Software, San Diego, CA, USA). Data are expressed as mean ± standard deviation (SD) derived from at least three independent replicate experiments. Normality of data distribution was confirmed via Shapiro–Wilk test (*α* = 0.05), and homogeneity of variance was verified using Bartlett’s test. For comparisons among multiple experimental groups versus the control group, one-way ANOVA with Sidak’s post hoc correction was applied to control type I error inflation in multiple testing. Statistical significance was established at *P* < 0.05.

## Supplementary information

Supplementary Figures, Methods and Appendix

Full and uncropped western blots

## Data Availability

The data supporting the findings of this study are available from the corresponding author upon reasonable request.
