# Supplementary material for: UVB-Induced necroptosis of the skin cells via RIPK3-MLKL activation independent of RIPK1 kinase activity
Source: Cell Death Discov. 2025 Apr 12;11:167. doi: 10.1038/s41420-025-02471-3 (PMC11993685; doi:10.1038/s41420-025-02471-3)
Supplement: Supplementary file 1 — Supplementary Figures, Methods and Appendix [file 41420_2025_2471_MOESM1_ESM.pdf]

Supplementary Figures

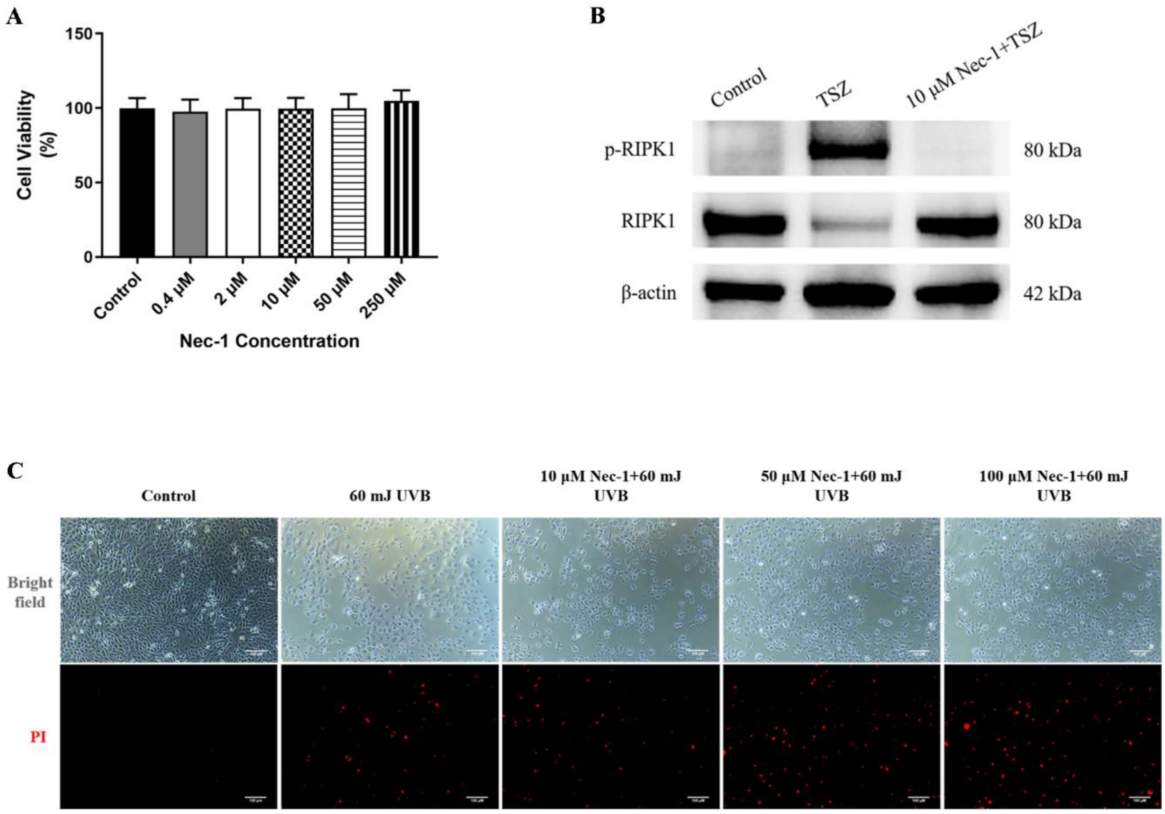

Fig. S1. Nec-1 treatment fails to rescue UVB-induced cell death. (A) Cell viability of HaCaT cells treated with varying concentrations of Nec-1 for 48 h, assessed using the CCK-8 assay. (B) Western blot analysis of pRIPK1 and RIPK1 in HaCaT cells 24 h after TSZ treatment or co-treatment with 10 μM Nec-1 and TSZ. (C) PI fluorescent staining of HaCaT cells and different concentrations of Nec-1 pretreated HaCaT cells 24 h after 60 mJ/cm^2 UVB irradiation; red staining indicating dead cells only (scale bar: 100 μm). Data are presented as mean ± SD, n=3. P values determined by one-way analysis of variance (ANOVA), $*P < 0.05$, $**P < 0.01$.

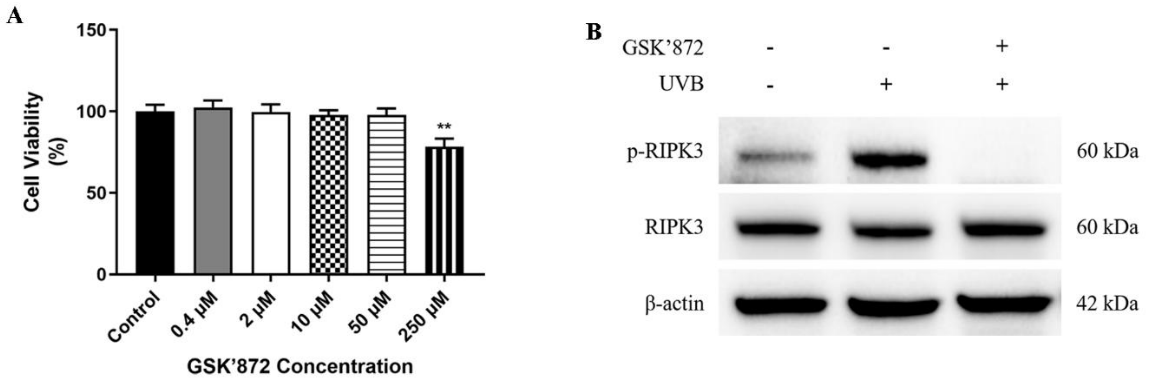

Fig. S2. 10 μM GSK'872 significantly inhibits UVB-induced pRIPK3 level without inhibiting HaCaT cell viability. (A) Cell viability of HaCaT cells treated with varying concentrations of GSK'872 for 48 h, assessed using the CCK-8 assay. (B) Western blot analysis of pRIPK3 and RIPK3 in HaCaT cells or 10 μM GSK'872 pretreated HaCaT cells 12 h after 60 mJ/cm^2 UVB irradiation. Data are presented as mean ± SD, n=3. P values determined by one-way analysis of variance (ANOVA), $*P < 0.05$, $**P < 0.01$.

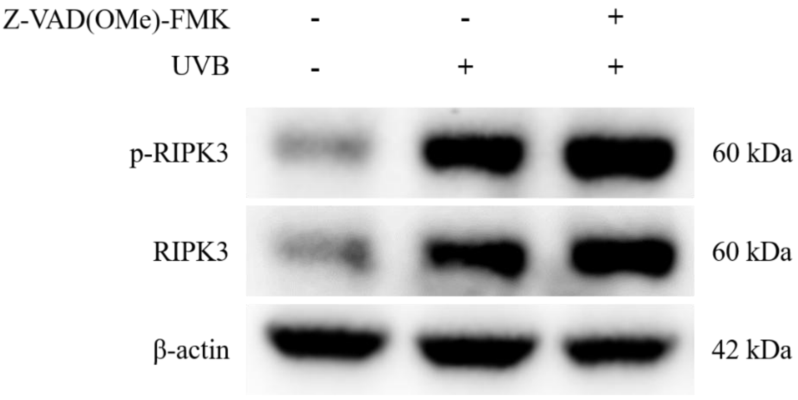

Fig. S3. Inhibition of caspase fails to affect UVB-induced phosphorylation of RIPK3. Western blot analysis of pRIPK3 and RIPK3 in HaCaT cells or 20 μM Z-VAD(OMe)-FMK pretreated HaCaT cells 24 h after 60 mJ/cm^2 UVB irradiation.

Supplementary Materials and Methods

Cell viability assay by CCK-8

HaCaT cells were seeded in 96-well plates, with wells containing no cells serving as blanks. The cells were then subjected to the appropriate treatments, including varying doses of UVB irradiation or different concentrations of drug incubation. After treatment, 10 μL of CCK-8 solution was added to each well, mixed thoroughly, and incubated for 1-4 hours until the color of the medium developed. Finally, the absorbance of each well was measured at 450 nm using a microplate reader, and cell viability was calculated based on the absorbance values.

Lentiviruses packaging

The negative control, RIPK1 and RIPK3 shRNA were cloned into the lentiviral vector LV2 (U6/Puro) as follows: Negative control shRNA sequence, 5’- TTCTCCGAACGTGTCACGT - 3’; RIPK1 shRNA sequence, 5’- GCATTCAGATTGGAGCCTACA - 3’; RIPK3 shRNA sequence, 5’- GCGACCGCTCGTTAACATATA - 3’. The full-length RIPK3 (CCDS ID: CCDS9628.1) was cloned into the lentiviral vector LV6 (EF-1a/Puro). The lentiviral constructs were co-transfected with the helper vectors pGag/Pol, pRev, and pVSV-G into 293T packaging cells. Lentivirus particles were purified by ultracentrifugation after collecting the cell supernatants 72 hours post-transfection.

Appendix

Full-length RIPK3 coding sequence (CCDS9628.1; 1557 nt):

ATGTCGTGCGTCAAGTTATGGCCCAGCGGTGCCCCCGCCCCCTTGGTGTCCATCGAGGAACTG
GAGAACCAGGAGCTCGTCGGCAAAGGCGGGTTCGGCACAGTGTTCCGGGCGCAACATAGGA
AGTGGGGCTACGATGTGGCGGTCAAGATCGTAAACTCGAAGGCGATATCCAGGGAGGTCAAG
GCCATGGCAAGTCTGGATAACGAATTCGTGCTGCGCCTAGAAGGGGTTATCGAGAAGGTGAA
CTGGGACCAAGATCCCAAGCCGGCTCTGGTGACTAAATTCATGGAGAACGGCTCCTTGTCGG
GGCTGCTGCAGTCCCAGTGCCCTCGGCCCTGGCCGCTCCTTTGCCGCCTGCTGAAAGAAGTG
GTGCTTGGGATGTTTTACCTGCACGACCAGAACCCGGTGCTCCTGCACCGGGACCTCAAGCCA
TCCAACGTCCTGCTGGACCCAGAGCTGCACGTCAAGCTGGCAGATTTTGGCCTGTCCACATTT
CAGGGAGGCTCACAGTCAGGGACAGGGTCCGGGGAGCCAGGGGGCACCCTGGGCTACTTGG
CCCCAGAACTGTTTGTTAACGTAAACCGGAAGGCCTCCACAGCCAGTGACGTCTACAGCTTC
GGGATCCTAATGTGGGCAGTGCTTGCTGGAAGAGAAGTTGAGTTGCCAACCGAACCATCACT
CGTGTACGAAGCAGTGTGCAACAGGCAGAACCGGCCTTCATTGGCTGAGCTGCCCCAAGCCG
GGCCTGAGACTCCCGGCTTAGAAGGACTGAAGGAGCTAATGCAGCTCTGCTGGAGCAGTGAG
CCCAAGGACAGACCCTCCTTCCAGGAATGCCTACCAAAAACTGATGAAGTCTTCCAGATGGT
GGAGAACAATATGAATGCTGCTGTCTCCACGGTAAAGGATTTCCTGTCTCAGCTCAGGAGCAG
CAATAGGAGATTTTCTATCCCAGAGTCAGGCCAAGGAGGGACAGAAATGGATGGCTTTAGGA
GAACCATAGAAAACCAGCACTCTCGTAATGATGTCATGGTTTCTGAGTGGCTAAACAAACTGA
ATCTAGAGGAGCCTCCCAGCTCTGTTCCTAAAAAATGCCCGAGCCTTACCAAGAGGAGCAGG
GCACAAGAGGAGCAGGTTCCACAAGCCTGGACAGCAGGCACATCTTCAGATTCGATGGCCCA
ACCTCCCCAGACTCCAGAGACCTCAACTTTCAGAAACCAGATGCCCAGCCCTACCTCAACTG
GAACACCAAGTCCTGGACCCCGAGGGAATCAGGGGGCTGAGAGACAAGGCATGAACTGGTC
CTGCAGGACCCCGGAGCCAAATCCAGTAACAGGGCGACCGCTCGTTAACATATACAACTGCTC
TGGGGTGCAAGTTGGAGACAACAACTACTTGACTATGCAACAGACAACTGCCTTGCCCACAT
GGGGCTTGGCACCTTCGGGCAAGGGGAGGGGCTTGCAGCACCCCCCACCAGTAGGTTCGCAA
GAAGGCCCTAAAGATCCTGAAGCCTGGAGCAGGCCACAGGGTTGGTATAATCATAGCGGGAA
ATAA
