# Supplementary material for: UVB-Induced necroptosis of the skin cells via RIPK3-MLKL activation independent of RIPK1 kinase activity
Source: Cell Death Discov. 2025 Apr 12;11:167. doi: 10.1038/s41420-025-02471-3 (PMC11993685; doi:10.1038/s41420-025-02471-3)

Original data of western blot

Fig. 1B. RIPK1

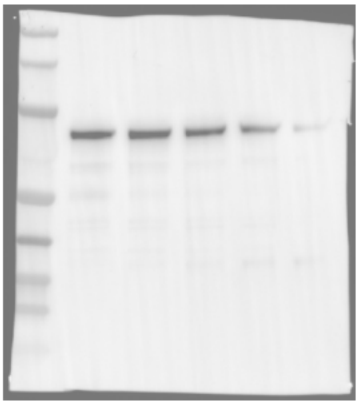

repeat:

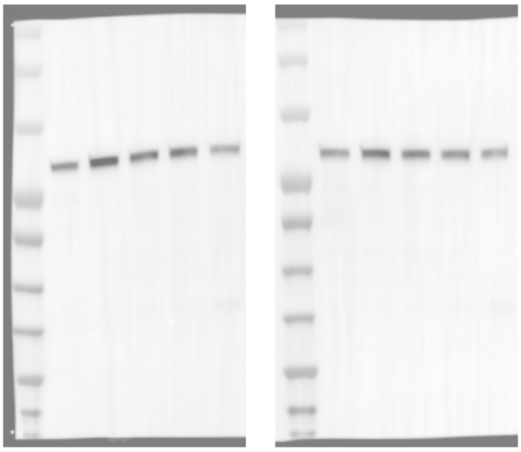

Fig. 1B. β-actin

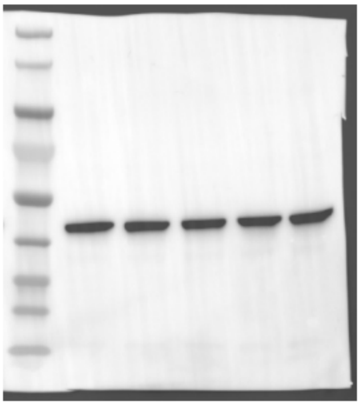

repeat:

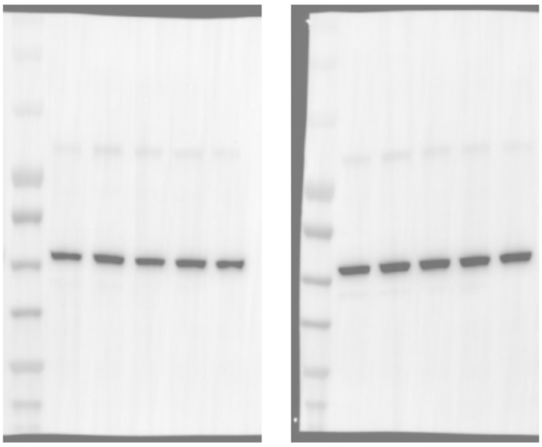

Fig. 1C. IκBα

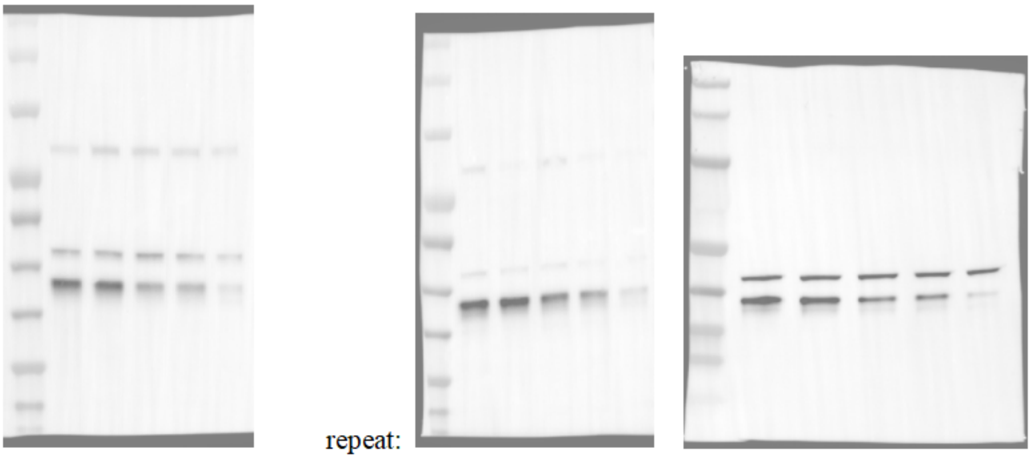

Fig. 1C. β-actin

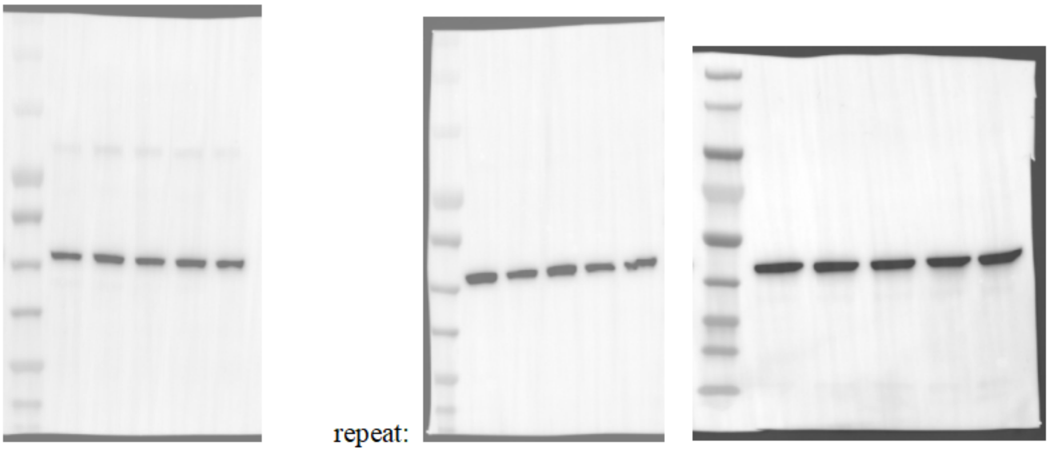

Fig. 1F. IκBα

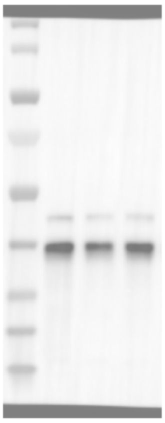

repeat:

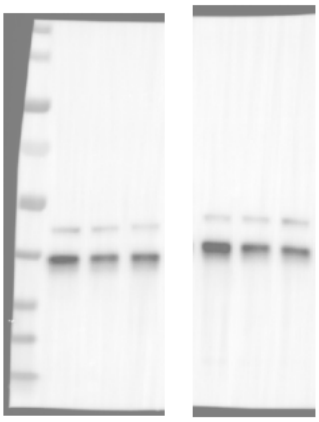

Figure 1F. RIPK1

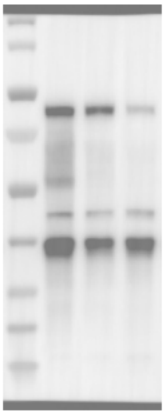

repeat:

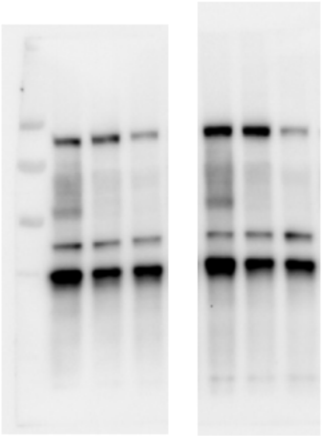

Figure 1F. β-actin

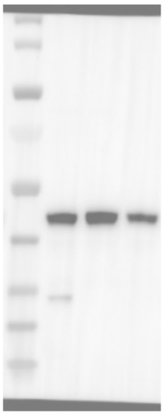

repeat:

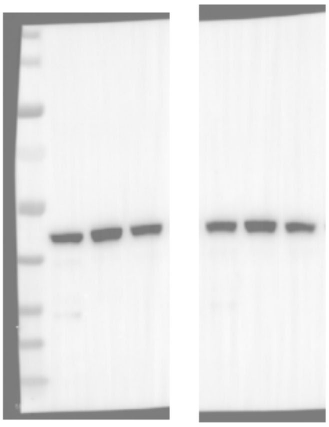

Figure 1G. p-p65

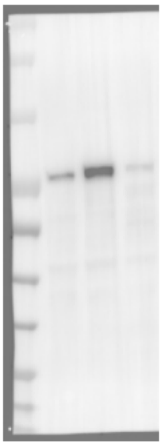

repeat:

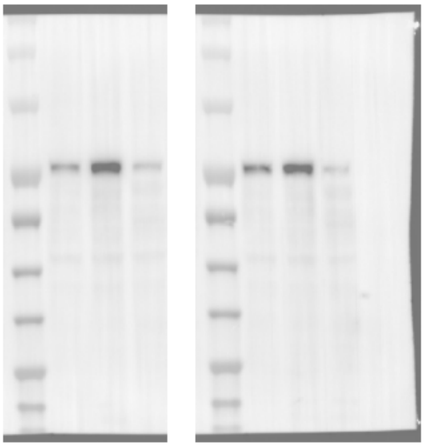

Figure 1G. p65

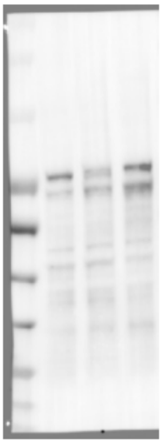

repeat:

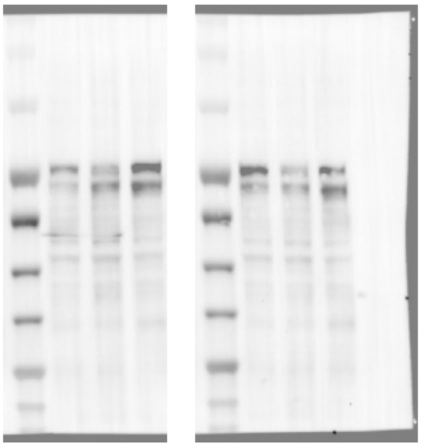

Figure 1G. β-actin

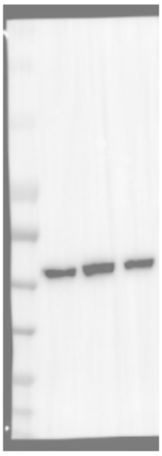

repeat:

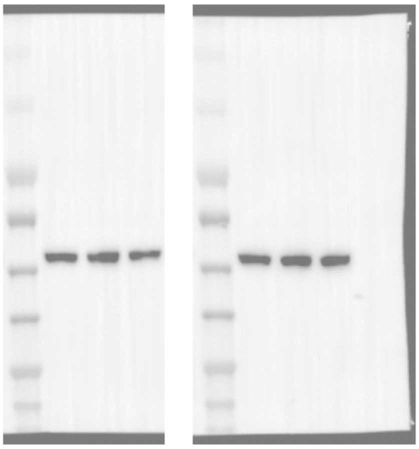

Fig. 2F. p-RIPK1 (0.5 h)

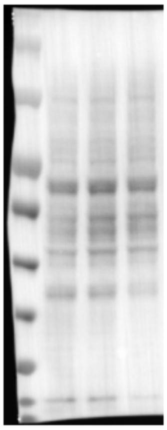

repeat:

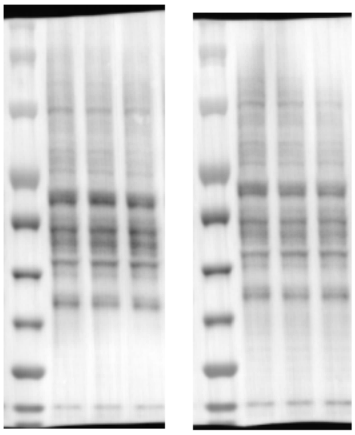

Fig. 2F. RIPK1 (0.5 h)

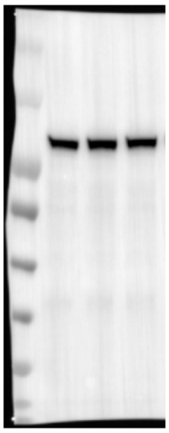

repeat:

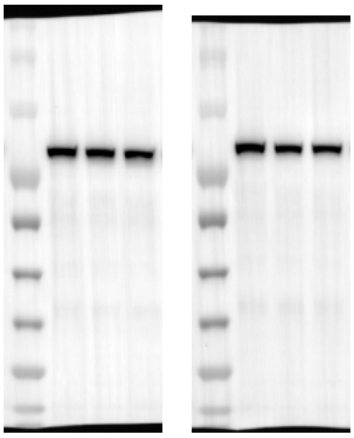

Fig. 2F. β-actin (0.5 h)

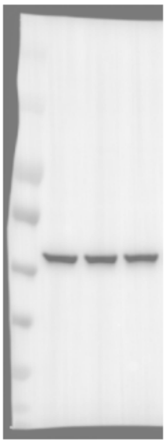

repeat:

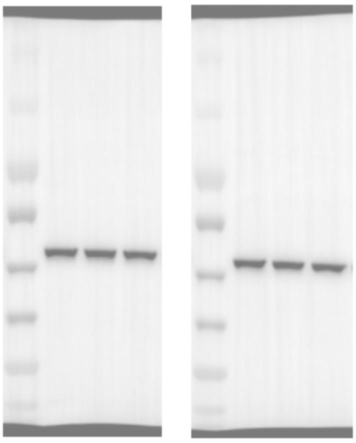

Fig. 2F. p-RIPK1 (3 h)

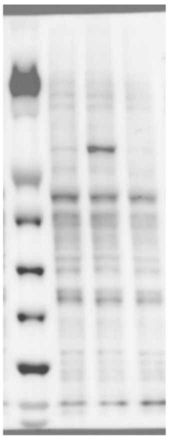

repeat:

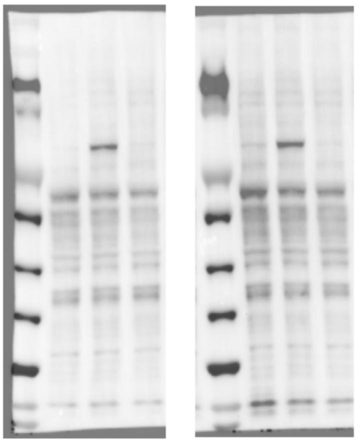

Fig. 2F. RIPK1 (3 h)

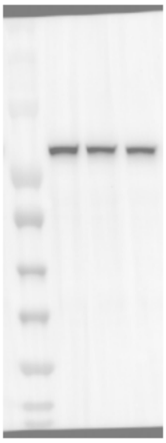

repeat:

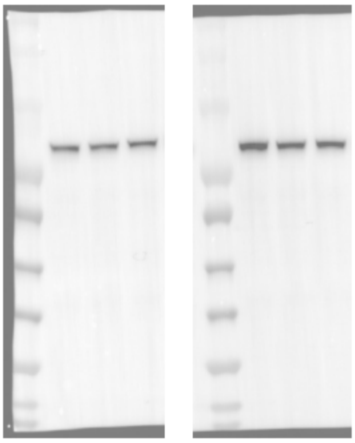

Fig. 2F. β-actin (3 h)

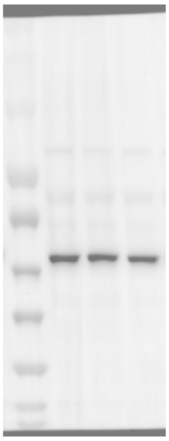

repeat:

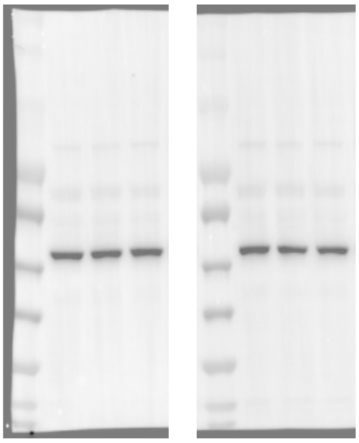

Fig. 2F. p-RIPK1 (6 h)

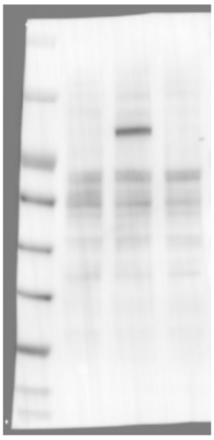

repeat:

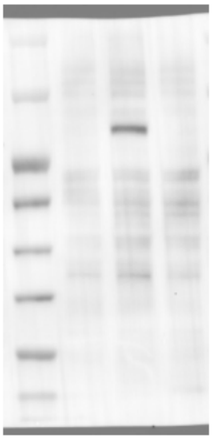

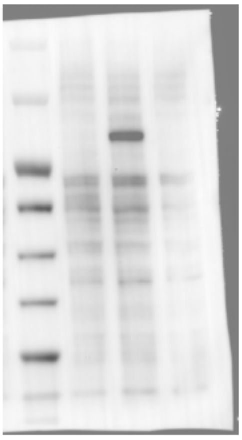

Fig. 2F. RIPK1 (6 h)

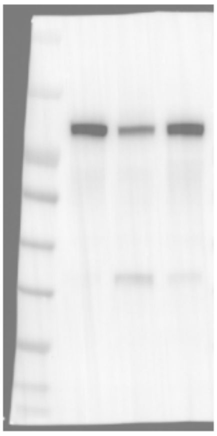

repeat:

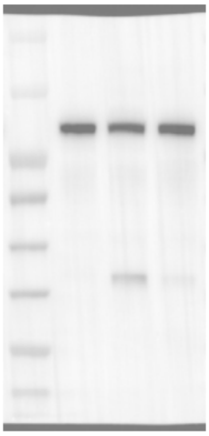

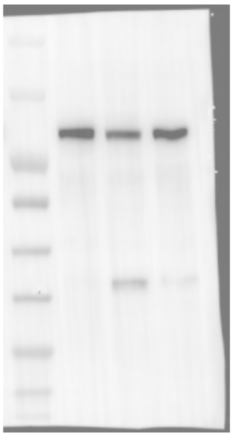

Fig. 2F. β-actin (6 h)

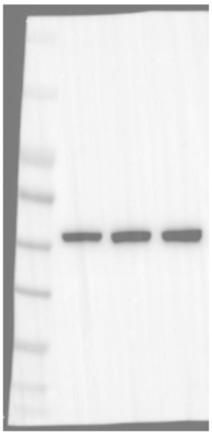

repeat:

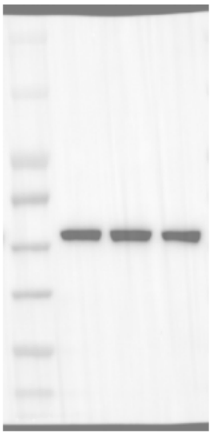

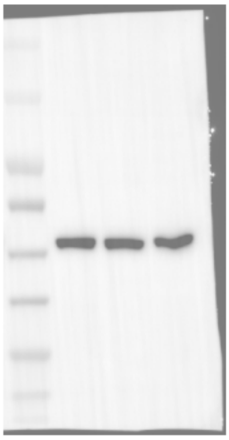

Fig. 2F. p-RIPK1 (12 h)

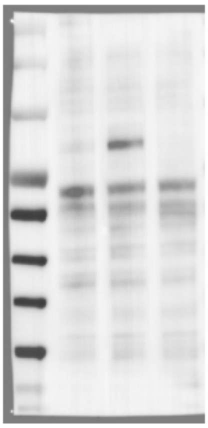

repeat:

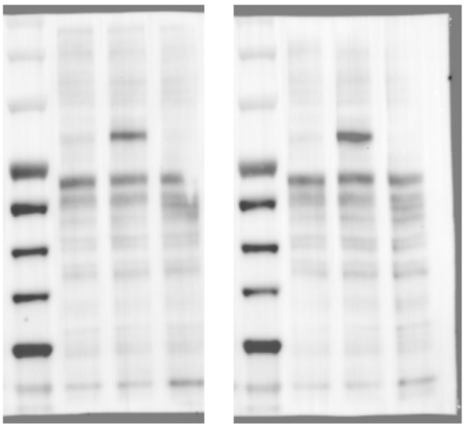

Fig. 2F. RIPK1 (12 h)

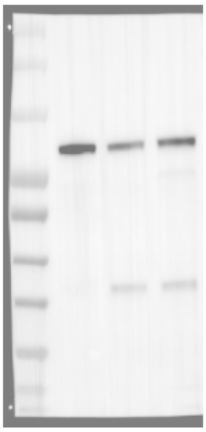

repeat:

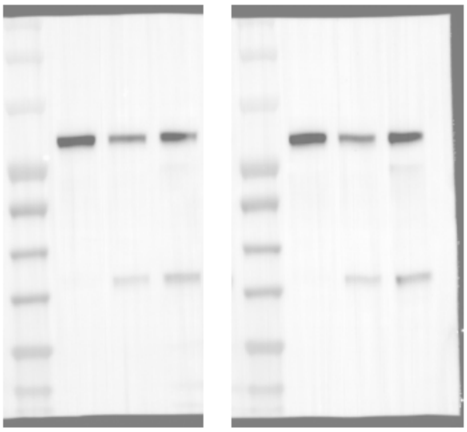

Fig. 2F. β-actin (12 h)

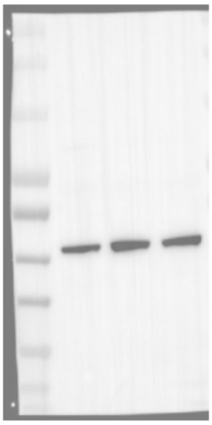

repeat:

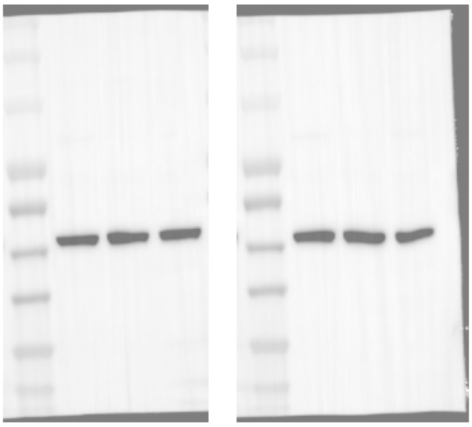

Fig. 2F. p-RIPK1 (24 h)

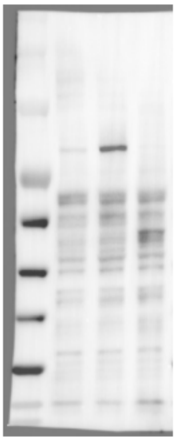

repeat:

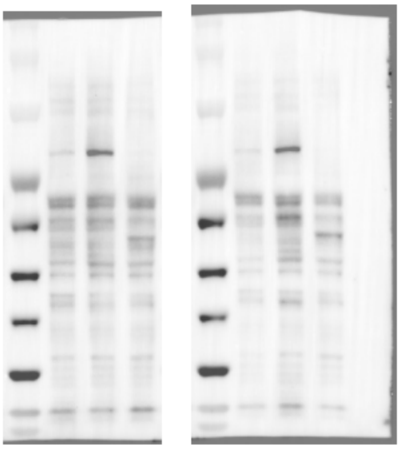

Fig. 2F. RIPK1 (24 h)

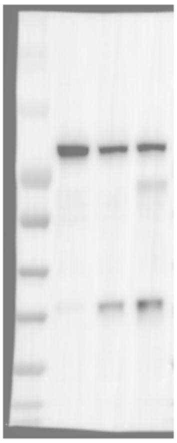

repeat:

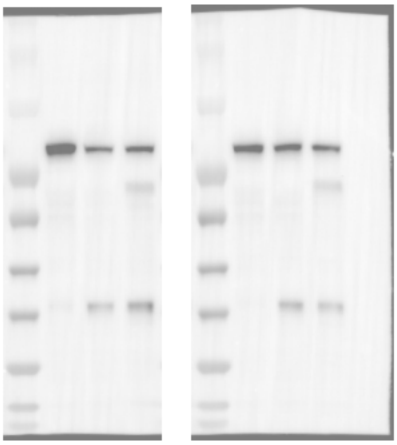

Fig. 2F. β-actin (24 h)

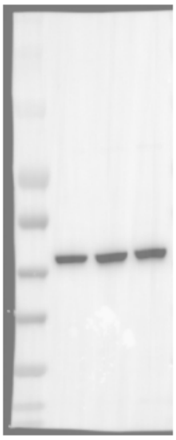

repeat:

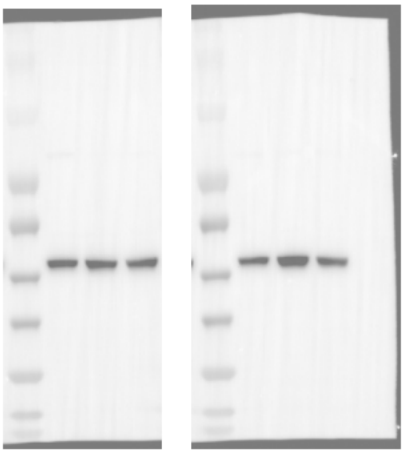

Fig. 3A. p-RIPK3 (3 h)

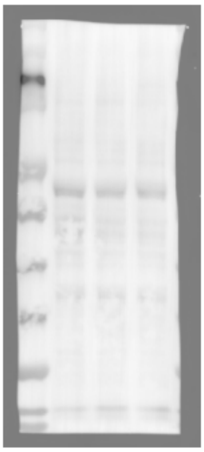

repeat:

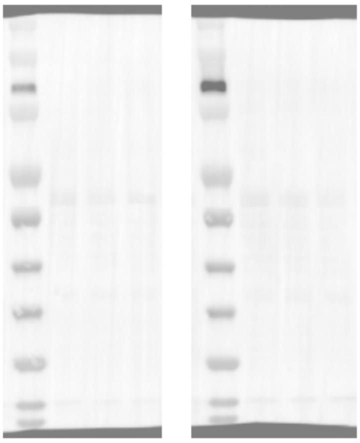

Fig. 3A. RIPK3 (3 h)

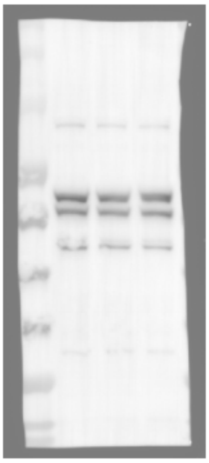

repeat:

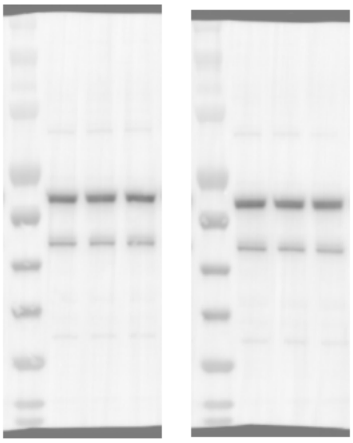

Fig. 3A. β-actin (3 h)

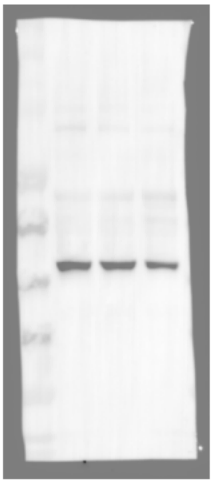

repeat:

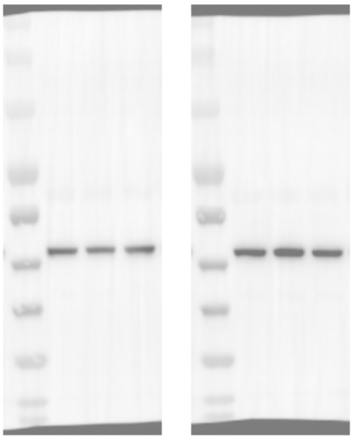

Fig. 3A. p-RIPK3 (6 h)

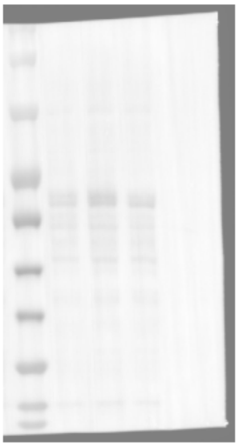

repeat:

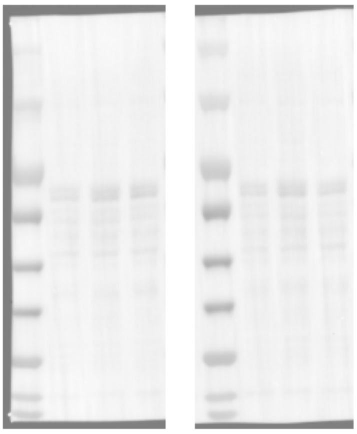

Fig. 3A. RIPK3 (6 h)

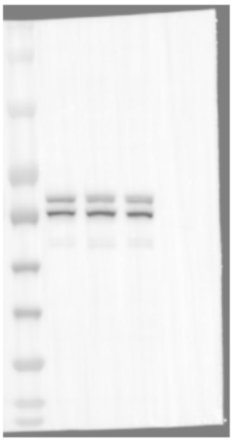

repeat:

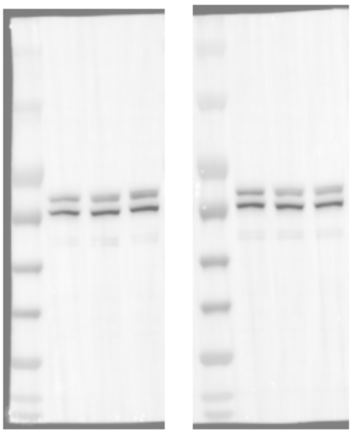

Fig. 3A. β-actin (6 h)

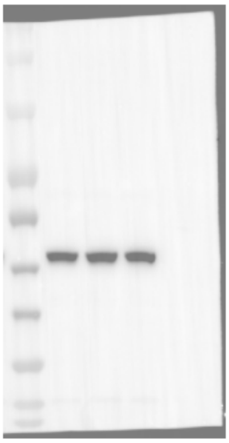

repeat:

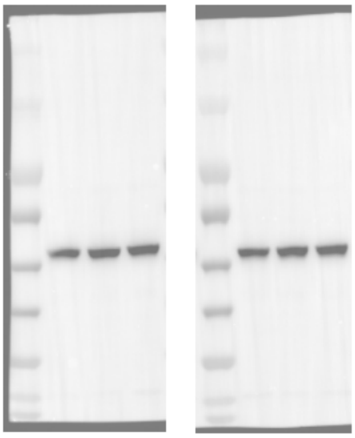

Fig. 3A. p-RIPK3 (12 h)

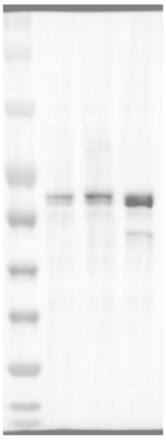

repeat:

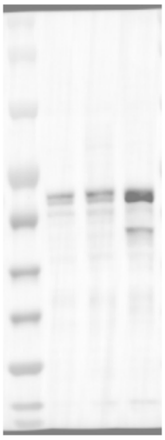

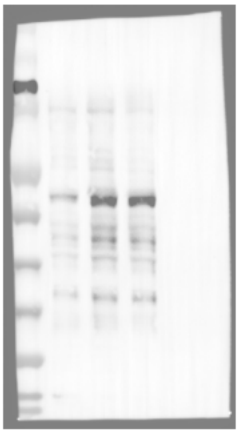

Fig. 3A. RIPK3 (12 h)

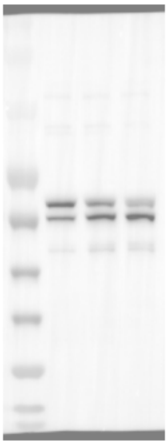

repeat:

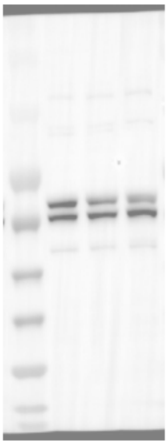

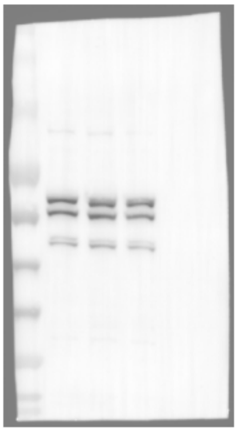

Fig. 3A. β-actin (12 h)

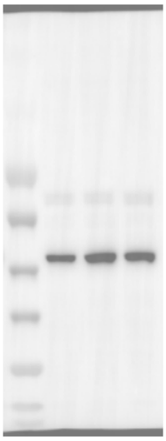

repeat:

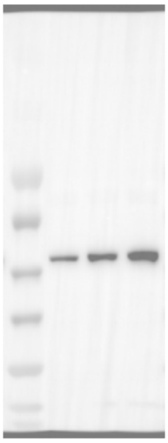

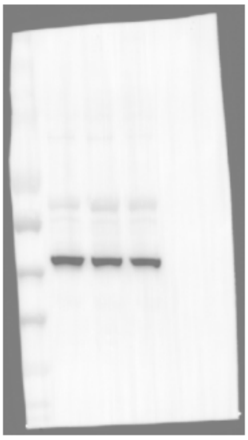

Fig. 3A. p-RIPK3 (24 h)

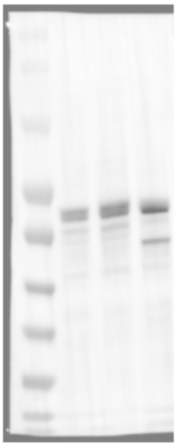

repeat:

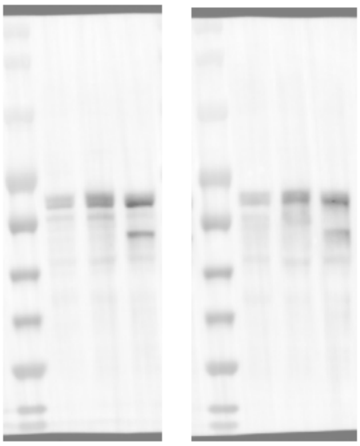

Fig. 3A. RIPK3 (24 h)

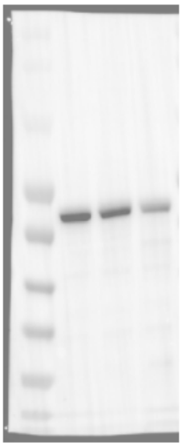

repeat:

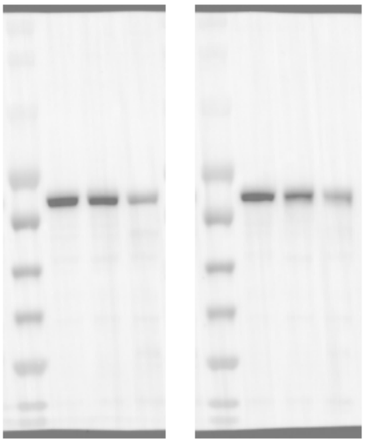

Fig. 3A. β-actin (24 h)

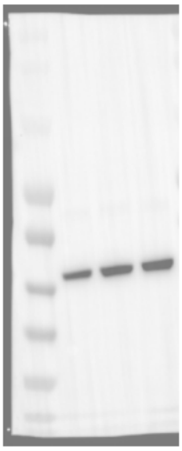

repeat:

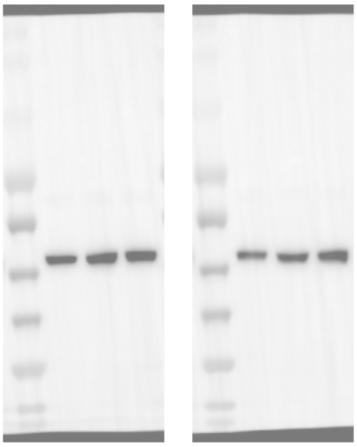

Fig. 3B. p-MLKL (3 h)

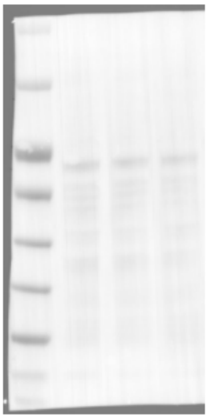

repeat:

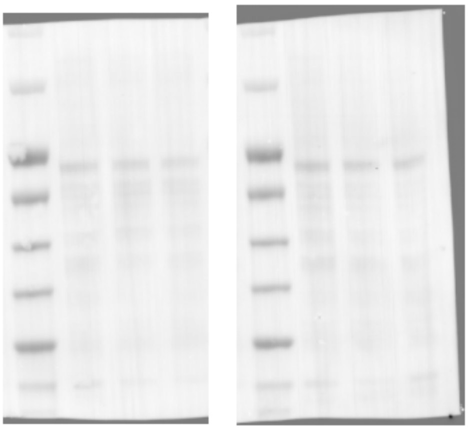

Fig. 3B. MLKL (3 h)

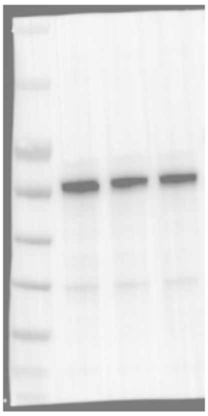

repeat:

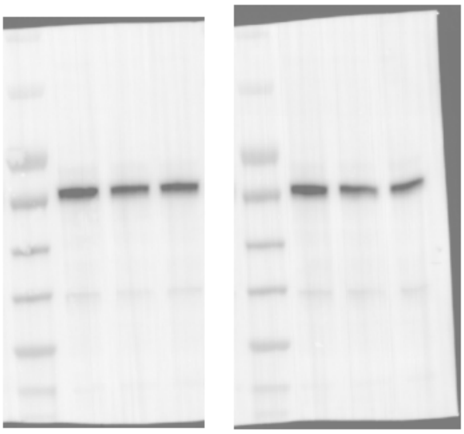

Fig. 3B. β-actin (3 h)

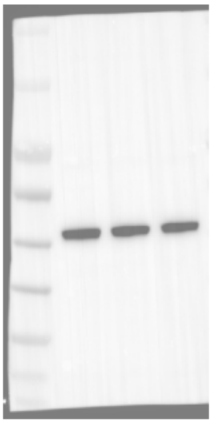

repeat:

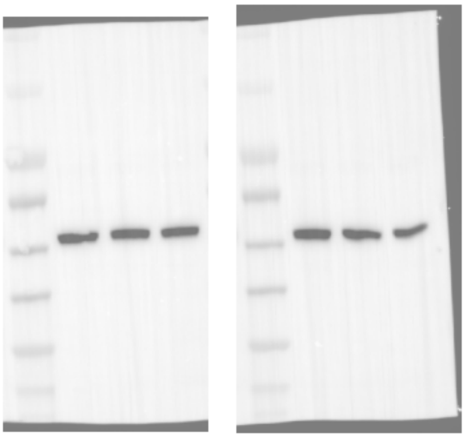

Fig. 3B. p-MLKL (6 h)

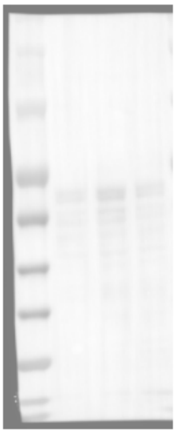

repeat:

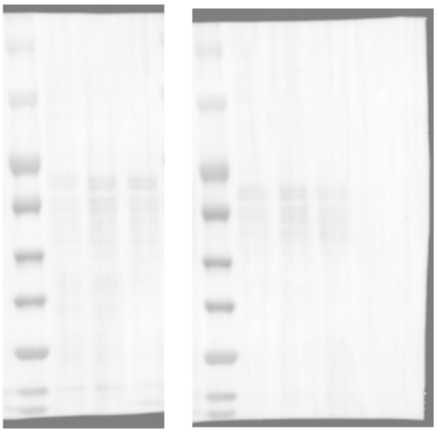

Fig. 3B. MLKL (6 h)

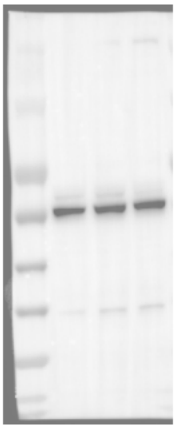

repeat:

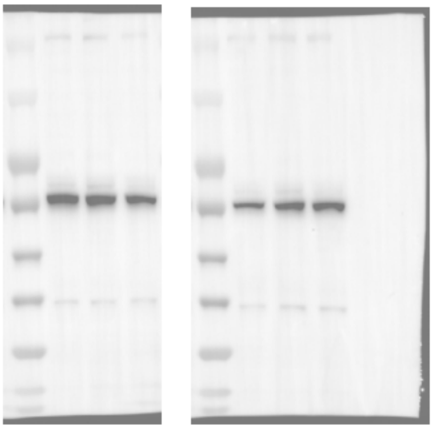

Fig. 3B. β-actin (6 h)

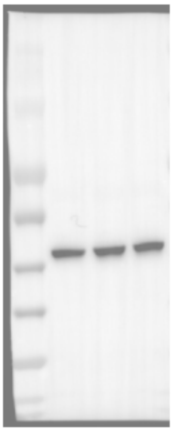

repeat:

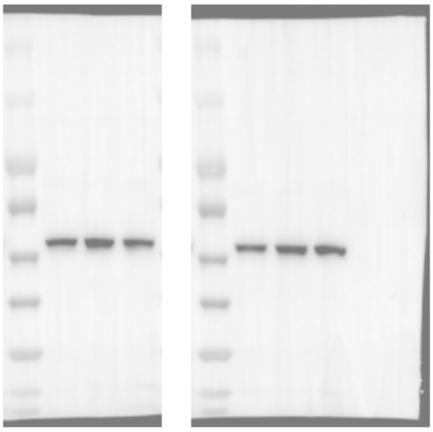

Fig. 3B. p-MLKL (12 h)

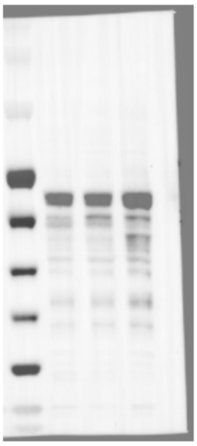

repeat:

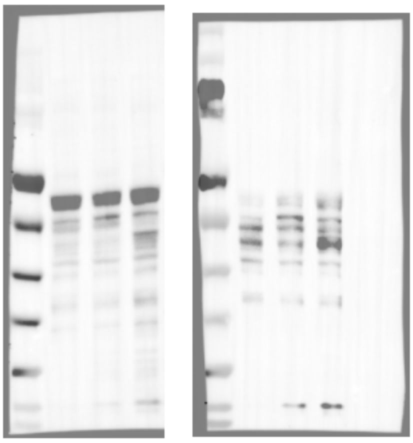

Fig. 3B. MLKL (12 h)

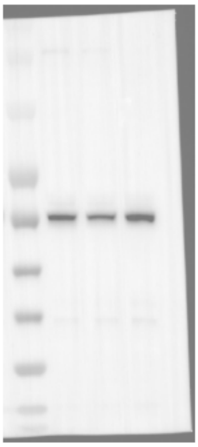

repeat:

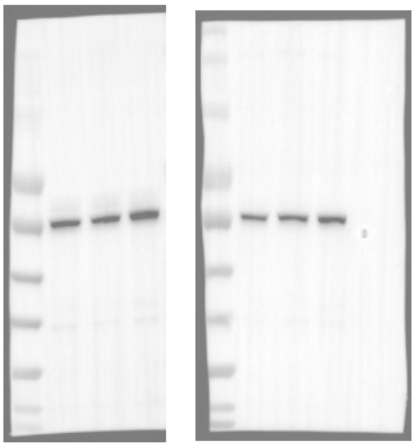

Fig. 3B. β-actin (12 h)

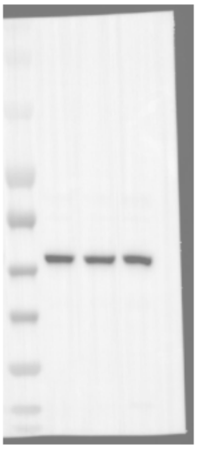

repeat:

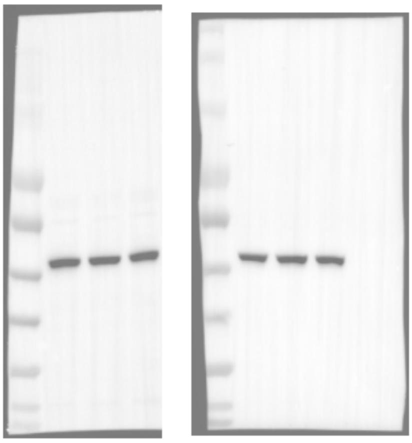

Fig. 3B. p-MLKL (24 h)

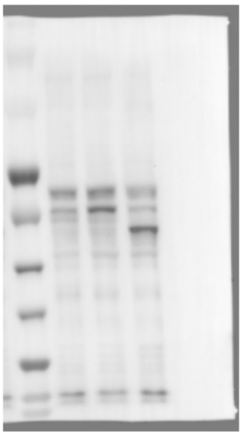

repeat:

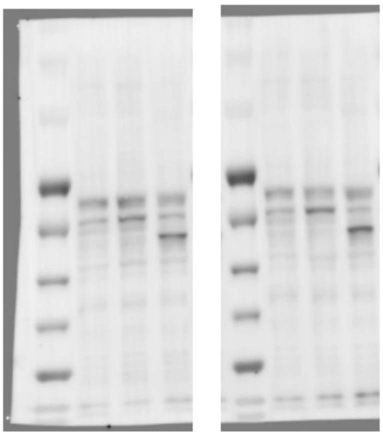

Fig. 3B. MLKL (24 h)

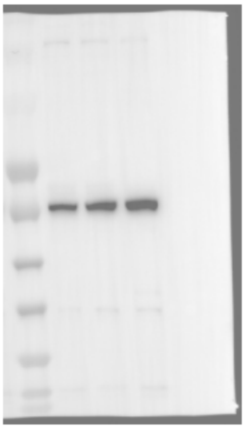

repeat:

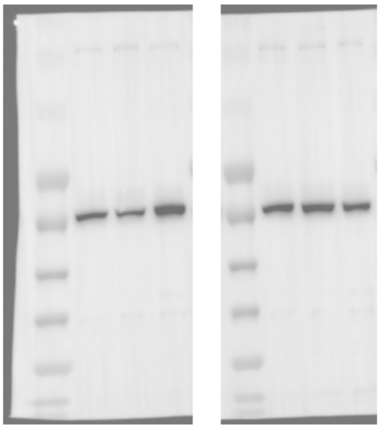

Fig. 3B. β-actin (24 h)

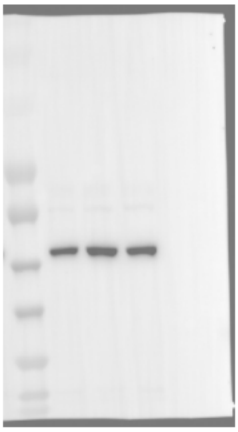

repeat:

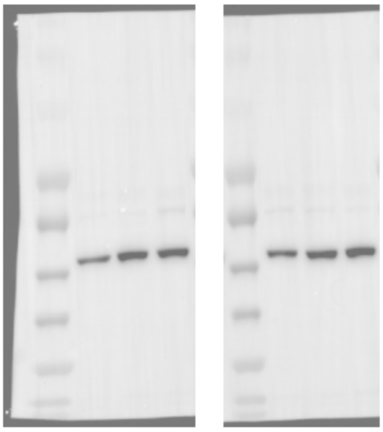

Fig. 4A. p-RIPK3

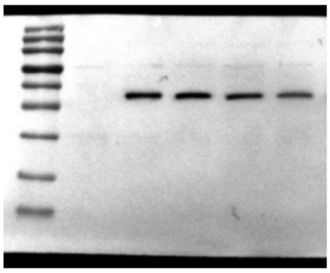

repeat:

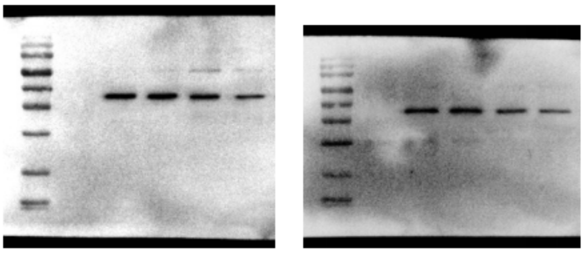

Fig. 4A. RIPK3

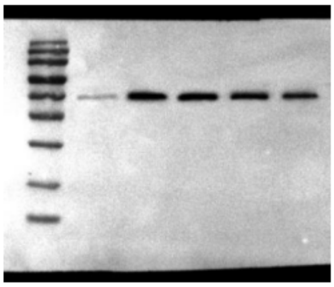

repeat:

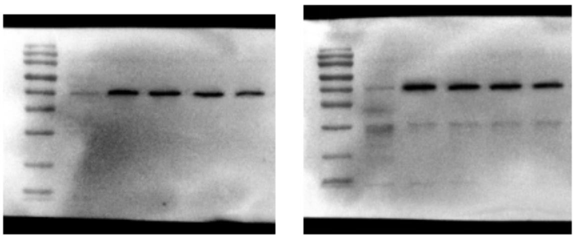

Fig. 4A. p-MLKL

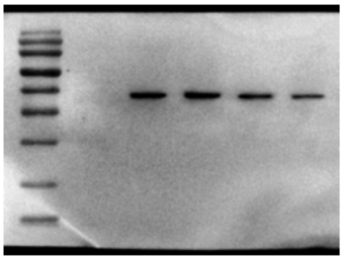

repeat:

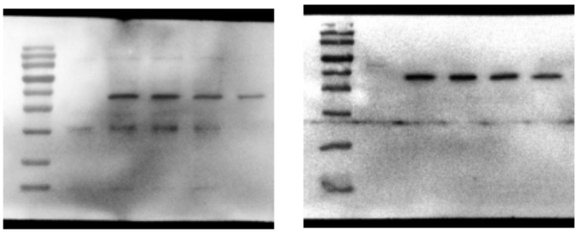

Fig. 4A. MLKL

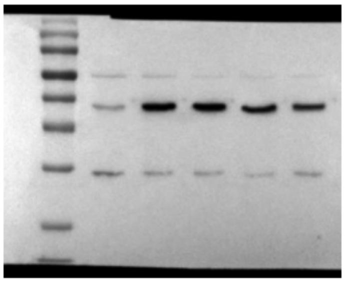

repeat:

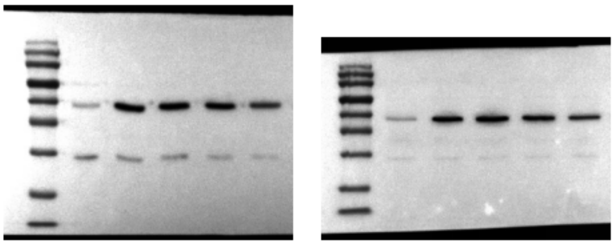

Fig. 4A. β-actin

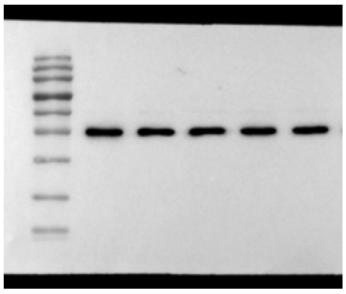

repeat:

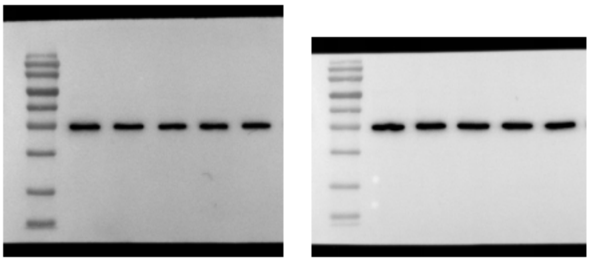

Fig. 4B. p-MLKL

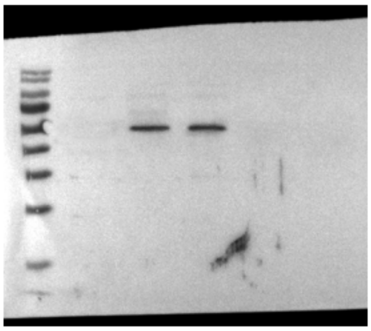

repeat:

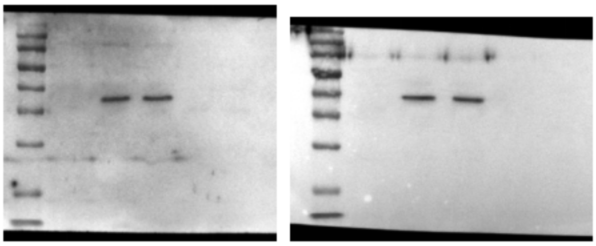

Fig. 4B. MLKL

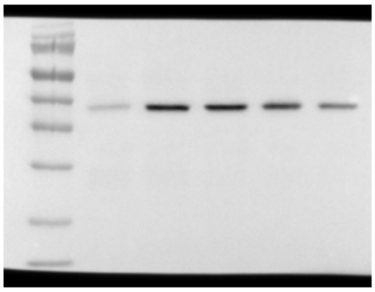

repeat:

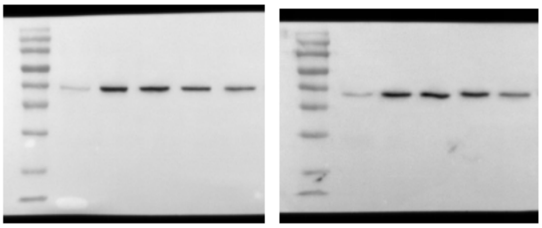

Fig. 4B. β-actin

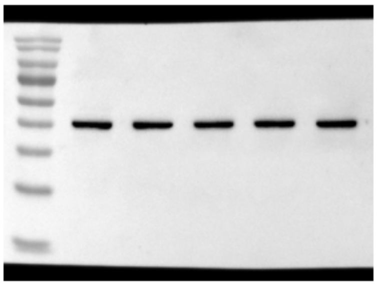

repeat:

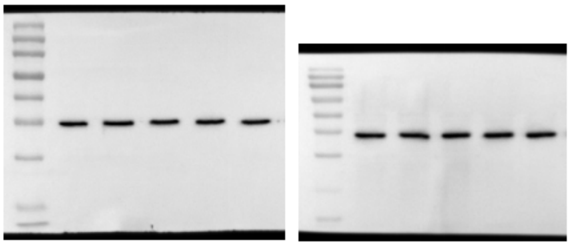

Fig. 4E. p-p65

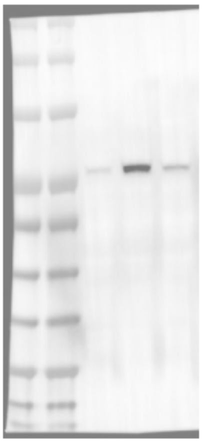

repeat:

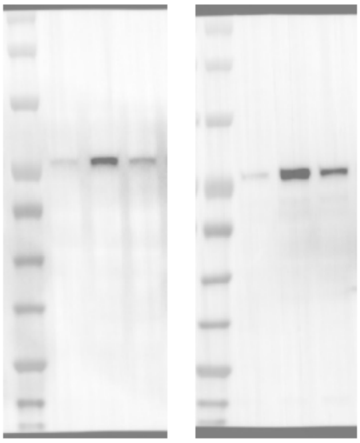

Fig. 4E. p65

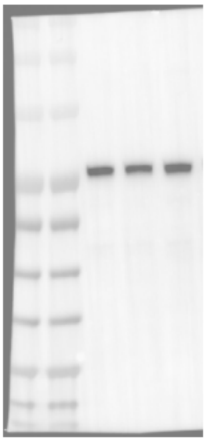

repeat:

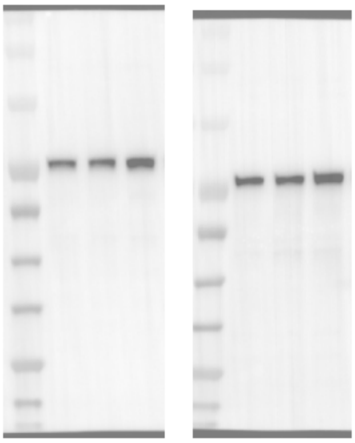

Fig. 4E. β-actin

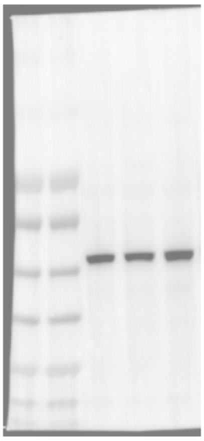

repeat:

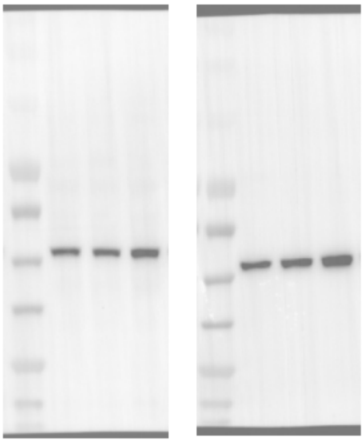

Fig. 5B. caspase-9 (full length and cleaved)

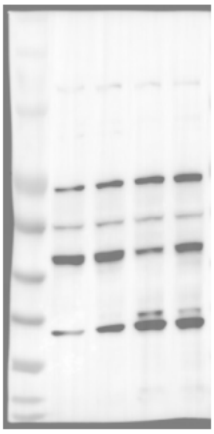

repeat:

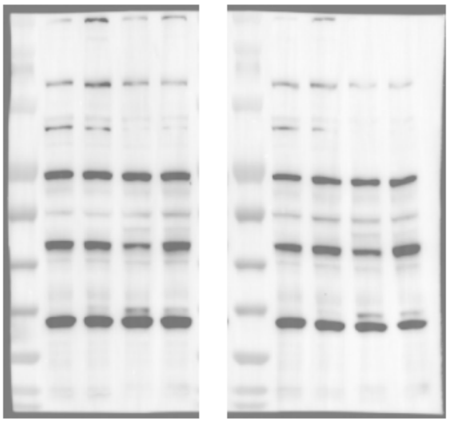

Fig. 5B. β-actin

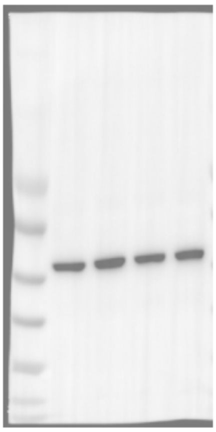

repeat:

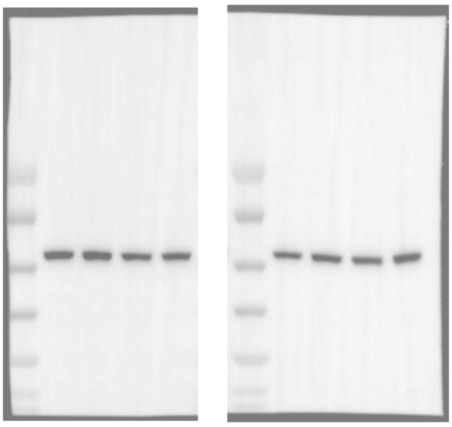

Fig. 5C. caspase-3 (full length and cleaved)

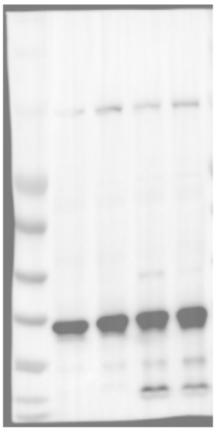

repeat:

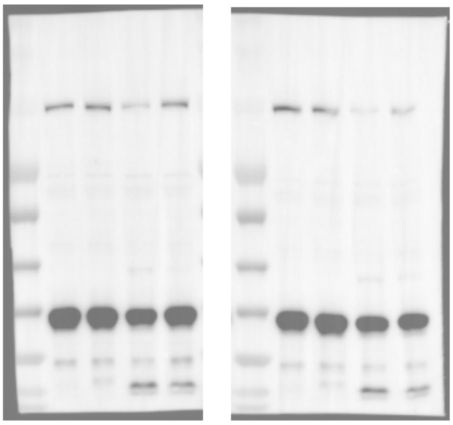

Fig. 5C. β-actin

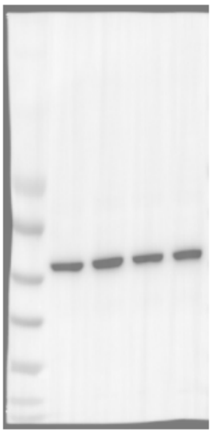

repeat:

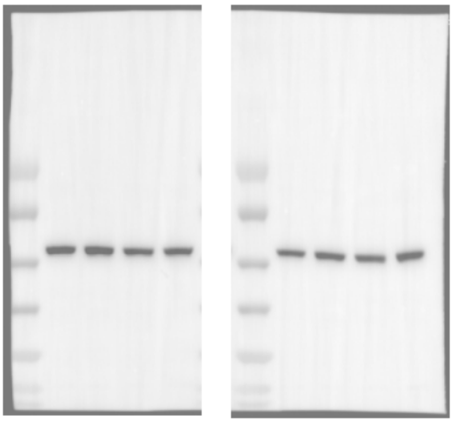

Fig. 5D. Bax

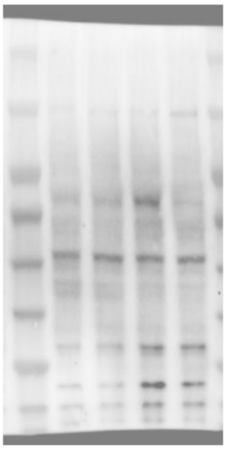

repeat:

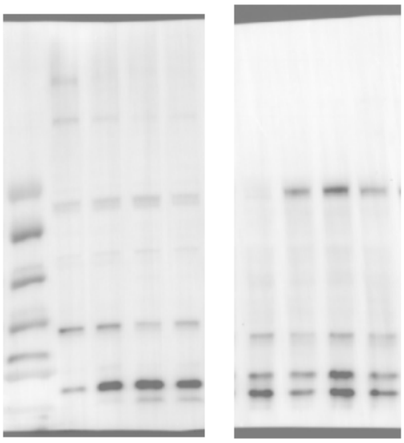

Fig. 5D. Bcl-2

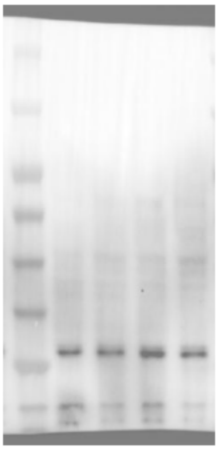

repeat:

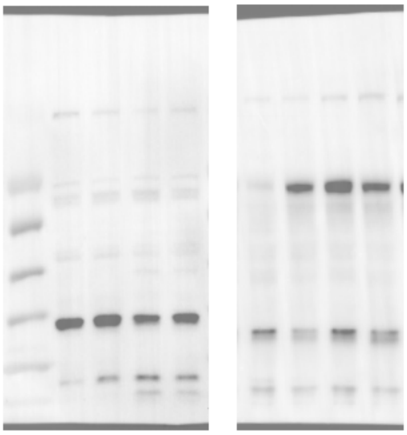

Fig. 5D. β-actin

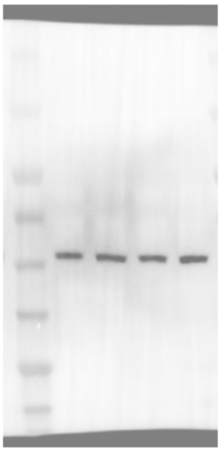

repeat:

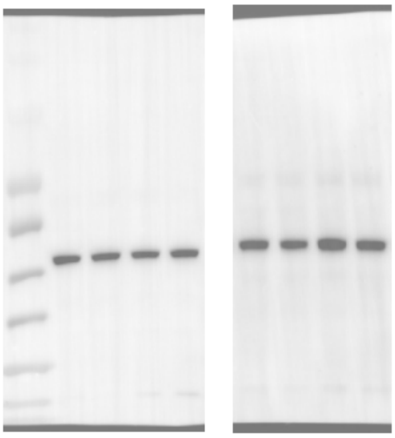

Fig. 6D. p-RIPK3

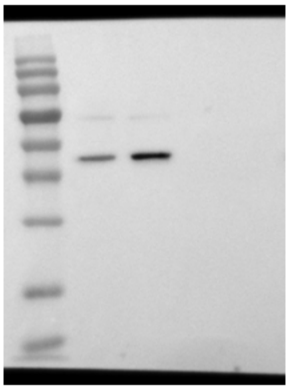

repeat:

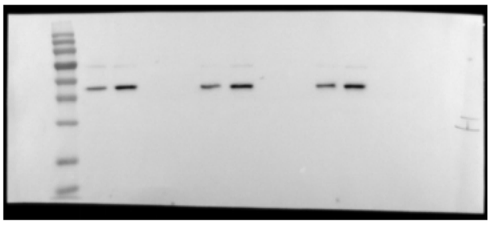

Fig. 6D. RIPK3

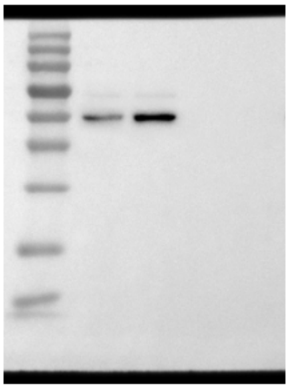

repeat:

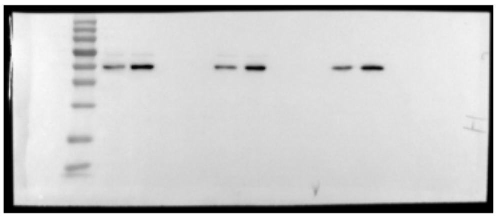

Fig. 6D. β-actin (RIPK3)

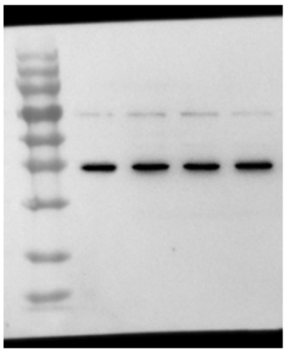

repeat:

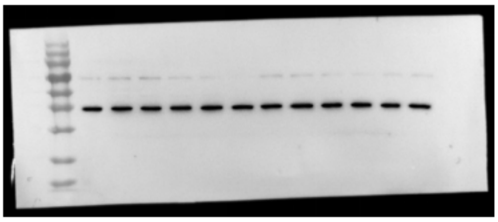

Fig. 6D. p-MLKL

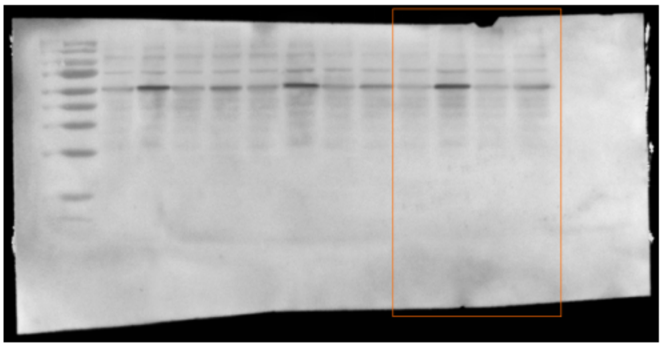

Fig. 6D. MLKL

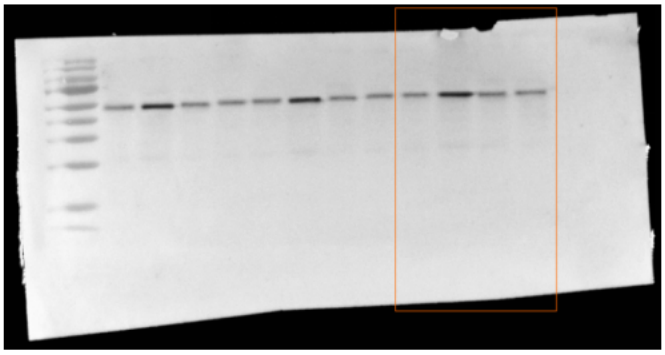

Fig. 6D. p-p65

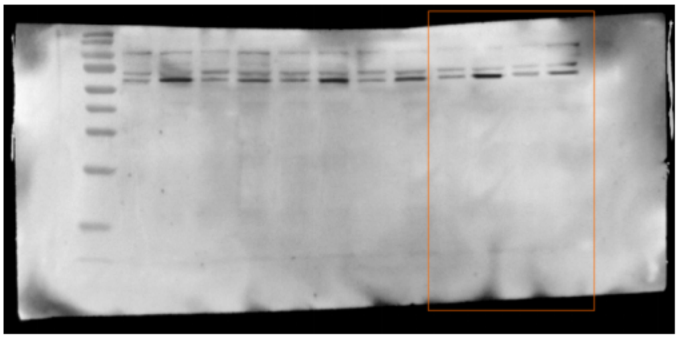

Fig. 6D. p65

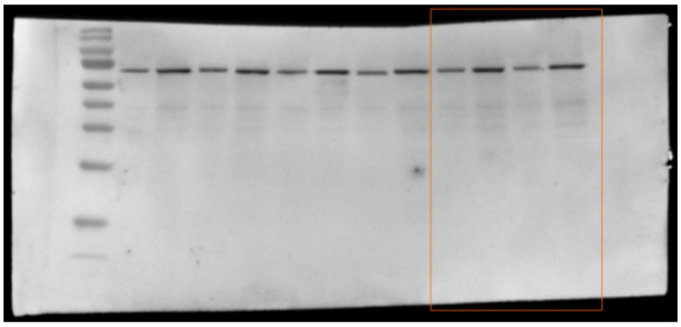

Fig. 6D. β-actin

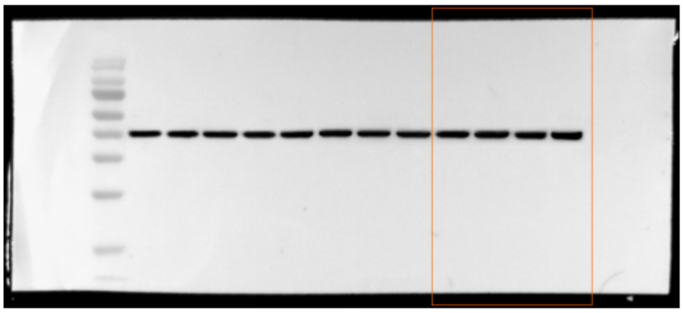

Supplement: Supplementary file 2 — Full and uncropped western blots [file 41420_2025_2471_MOESM2_ESM.pdf]
